# Heterologous Protection against Malaria after Immunization with *Plasmodium falciparum* Sporozoites

**DOI:** 10.1371/journal.pone.0124243

**Published:** 2015-05-01

**Authors:** Remko Schats, Else M. Bijker, Geert-Jan van Gemert, Wouter Graumans, Marga van de Vegte-Bolmer, Lisette van Lieshout, Mariëlle C. Haks, Cornelus C. Hermsen, Anja Scholzen, Leo G. Visser, Robert W. Sauerwein

**Affiliations:** 1 Leiden University Medical Center, Department of Infectious Diseases, PO Box 9600, 2300 RC, Leiden, The Netherlands; 2 Radboud university medical center, Department of Medical Microbiology, PO Box 9101, 6500 HB, Nijmegen, The Netherlands; 3 Leiden University Medical Center, Department of Medical Microbiology, PO Box 9600, 2300 RC, Leiden, The Netherlands; 4 Leiden University Medical Center, Department of Parasitology, PO Box 9600, 2300 RC, Leiden, The Netherlands; Centers for Disease Control and Prevention, UNITED STATES

## Abstract

**Background:**

Sterile protection in >90% of volunteers against homologous *Plasmodium falciparum* infection has been achieved only using the controlled human malaria infection (CHMI) model. This efficient model involves whole parasite immunizations under chloroquine prophylaxis (CPS-immunization), requiring only 30–45 mosquitoes bites infected with *P*. *falciparum*-sporozoites. Given the large diversity of *P*. *falciparum* parasites, it is essential to assess protection against heterologous parasite strains.

**Methods:**

In an open-label follow-up study, 16 volunteers previously CPS-immunized and challenged with *P*. *falciparum* NF54 (West-Africa) in a dose de-escalation and challenge trial were re-challenged with clone NF135.C10 (Cambodia) at 14 months after the last immunization (NCT01660854).

**Results:**

Two out of thirteen NF54 protected volunteers previously fully protected against NF54 were also fully protected against NF135.C10, while 11/13 showed a delayed patency (median prepatent period of 10.5 days (range 9.0–15.5) versus 8.5 days in 5 malaria-naïve controls (p = 0.0005). Analysis of patency by qPCR indicated a 91 to >99% estimated reduction of liver parasite load in 7/11 partially protected subjects. Three volunteers previously not protected against NF54, were also not protected against NF135.C10.

**Conclusion:**

This study shows that CPS-immunization can induce heterologous protection for a period of more than one year, which is a further impetus for clinical development of whole parasite vaccines.

**Trial Registration:**

Clinicaltrials.gov NCT01660854

## Introduction

Malaria remains a tremendous public health problem affecting approximately 40% of the world’s population. The global incidence of malaria is estimated to be around 198 million clinical cases resulting in 584.000 deaths [1] most of which are caused by *P*. *falciparum*. Since current interventions fail to reduce malaria incidence sufficiently, a vaccine is urgently needed to combat this disease.

Sterile protection against *P*. *falciparum* malaria can efficiently and reproducibly be achieved in the Controlled Human Malaria Infection (CHMI) setting by repeated inoculation of live sporozoites by bites of laboratory-reared *Anopheles* mosquitoes to healthy malaria-naïve volunteers under chemoprophylaxis: ChemoProphylaxis and Sporozoites (CPS-) immunization [2,3]. CPS-induced protection is dose-dependent [3] and was shown in a subset of volunteers to last for more than two years [4]. Furthermore, bites from only 30–45 *P*. *falciparum*-infected mosquitoes are sufficient to induce sterile protection in >90% of subjects, while immunization with radiation-attenuated sporozoites (RAS) requires a minimum of 1,000 *P*. *falciparum*-infected mosquitoes, or intravenous injection of 675,000 cryopreserved sporozoites [5,6]. So far CPS-immunizations and challenges have been performed with the homologous NF54 strain only, while in malaria-endemic areas there is a large genetic and antigenic diversity of *P*. *falciparum* strains. This diversity is considered an important reason why naturally acquired immunity is obtained slowly, only after several years of repeated exposure [7]. Previously, heterologous protection has been reported in 4/6 RAS-immunized volunteers [5].

Next to the widely used *P*. *falciparum* strain NF54 and its clone 3D7, NF135.C10 originating from Cambodia has become available for CHMI studies [7]. In this study, volunteers who had previously participated in a NF54 dose de-escalation CPS-immunization and challenge trial were re-challenged with NF135.C10 after more than one year.

## Materials and Methods

The protocol for this trial and supporting TREND checklist are available as supporting information; see S1 Checklist and S1 Protocol.

### Study design

A single centre open label clinical trial was conducted at the Leiden University Medical Center (LUMC) from July 2012 until February 2013. The study was approved by the Central Committee for Research Involving Human Subjects of The Netherlands (NL39414.000.12) and complied with the Declaration of Helsinki and Good Clinical Practice including monitoring of data. ClinicalTrials.gov Identifier: NCT01660854.

### Study participants

**Fig 1:**
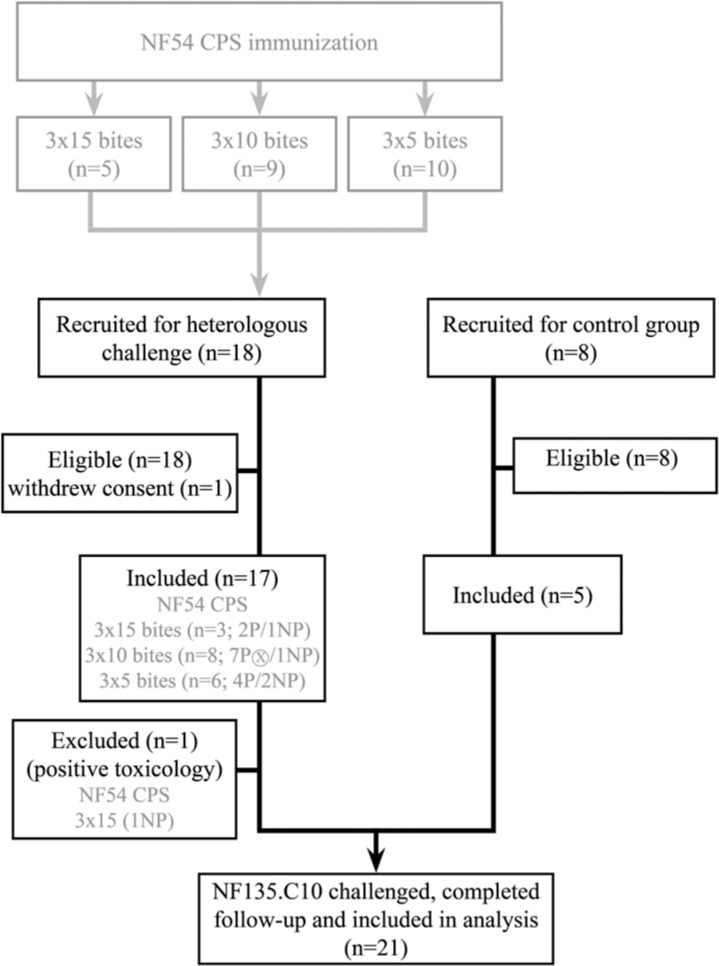
Study flow diagram. The previous NF54 CPS-immunization study is shown in grey. P = NF54 protected, NP = NF54 unprotected. ⨂ = Volunteer presumptively treated on day 10.5 after NF54 challenge and considered NF54 protected

Eighteen volunteers from a NF54 CPS dose-de-escalating study (ClinicalTrials.gov Identifier: NCT01218893;[8]) and 8 newly recruited malaria-naïve subjects aged 18–35 years were all screened in July 2012 for eligibility based on medical and family history, physical examination and standard haematological and biochemical measurements (**Fig 1**). Seventeen NF54 CPS-immunized volunteers and five controls were included. One volunteer had to be excluded because of a positive urine toxicology test for cannabis and was treated with atovaquone/proguanil two days after challenge. Two of the remaining included volunteers had previously received the highest dose of NF54 CPS (3x15 bites), 8 a medium dose (3x10 bites) and 6 the lowest dose (3x5 bites). Thirteen were NF54 protected, of which one volunteer was presumptively treated because of a non-malaria related SAE on day 10,5 after NF54 challenge but considered NF54 protected [8]. The sample size calculation for this study is described in detail in S1 Methods.

None of the female volunteers were pregnant or lactating. Serology for HIV, hepatitis B and hepatitis C was negative in all volunteers. Plasma samples tested by Enzyme-Linked ImmunoSorbent Assay (ELISA) against crude NF54 asexual blood stages were negative in all control volunteers. None of the volunteers had travelled to a malaria-endemic area within 6 months prior to the start of the study. All volunteers provided written informed consent before screening.

### Study procedures

All volunteers were challenged simultaneously by exposure to five bites of *Anopheles stephensi* mosquitoes infected with the NF135.C10 *P*. *falciparum* clone in August 2012 [9]. This heterologous challenge was performed 14 months after the last NF54 CPS-immunization and 9.5 months after NF54 challenge. Volunteers were followed-up on an outpatient basis once daily on days 5–6 after challenge, twice daily between days 7–15 and once daily between days 16–21. During each visit, blood was drawn for parasite detection by thick smear. Volunteers were treated with 1000 mg atovaquone and 400 mg proguanil once daily for three days according to Dutch national malaria guidelines as soon as parasites were detected by thick smear, or on day 21 after challenge if they had remained thick smear negative. The last visit for volunteers was conducted in February 2013.

Safety parameters were determined daily: platelet counts were determined in EDTA blood with the Sysmex XE-2100 (Sysmex Europe GmbH. Norderstedt. Germany). D-dimer concentrations were assessed in citrate plasma by STA-R Evolution (Roche Diagnostics, Almere, The Netherlands; upper limit of detection 5000 ng/ml), Highly sensitive (Hs) Troponine T and Lactate Hydrogenase (LDH) were determined in serum by Modular E170 (Roche Diagnostics, Almere, The Netherlands).

### Endpoints

The primary endpoint was time to parasitemia after challenge infection as assessed by thick smear. Blood was screened by microscopy for parasites as described before, and the thick smear was considered positive if two unambiguous parasites were detected in 0.5μL of blood, confirmed by a second independent reader. Volunteers were considered protected when thick smear remained negative up until 21 days after challenge.

Secondary endpoints were the kinetics of parasitemia and frequency of signs and symptoms. Parasitemia was retrospectively quantified by qPCR on samples collected up to twice daily from day 5 until day 21 after challenge as described previously [10] with some modifications. Briefly, 5μL Zap-oglobin II Lytic Reagent (Beckman Coulter) was added to 0.5ml of EDTA blood, after which the samples were mixed and stored at -80°C. After thawing, samples were spiked with the extraction control Phocine Herpes Virus (PhHV) and DNA was extracted with a MagnaPure LC isolation station. Isolated DNA was resuspended in 50μl H_2_O and 5μl was used as template. For the detection of *P*. *falciparum*, the primers as described earlier [10] and the TaqMan MGB FAM-labelled probe 5’-AACAATTGGAGGGCAAG-3’ were used. For quantification of PhHV the primers 5’-GGGCGAATCACAGATTGAATC-3’, 5’-GCGGTTCCAAACGTACCAA-3’ and the probe Cy5-5’-TTTTTATGTGTCCGCCACCATCTGGATC-3’ were used.

Adverse events (AE) reported by volunteers or observed by the investigator were recorded according to the following scale: *mild* (grade 1; easily tolerated), *moderate* (grade 2; interferes with normal activity) or *severe* (grade 3; prevents normal activity). Fever was recorded as grade 1 (37.5–38.0°C), grade 2 (38.0–39.0°C) or grade 3 (>39.0°C).

### Statistical analysis

All possibly and probably (both solicited and unsolicited) related AE were tabulated, grouped and analysed by calculating the average number of mild, moderate or severe AE per volunteer in each group. Statistical analyses were performed using GraphPad Prism 6.02. Differences in prepatent period and parasitemia at time of treatment between two groups (NF54 protected and controls) were tested by Mann Whitney test, and between the three dose groups by Kruskal-Wallis test with Dunn’s multiple comparison post-hoc test. A p value of <0.05 was considered statistically significant.

## Results

### Heterologous protection induced by CPS-immunization

**Fig 2:**
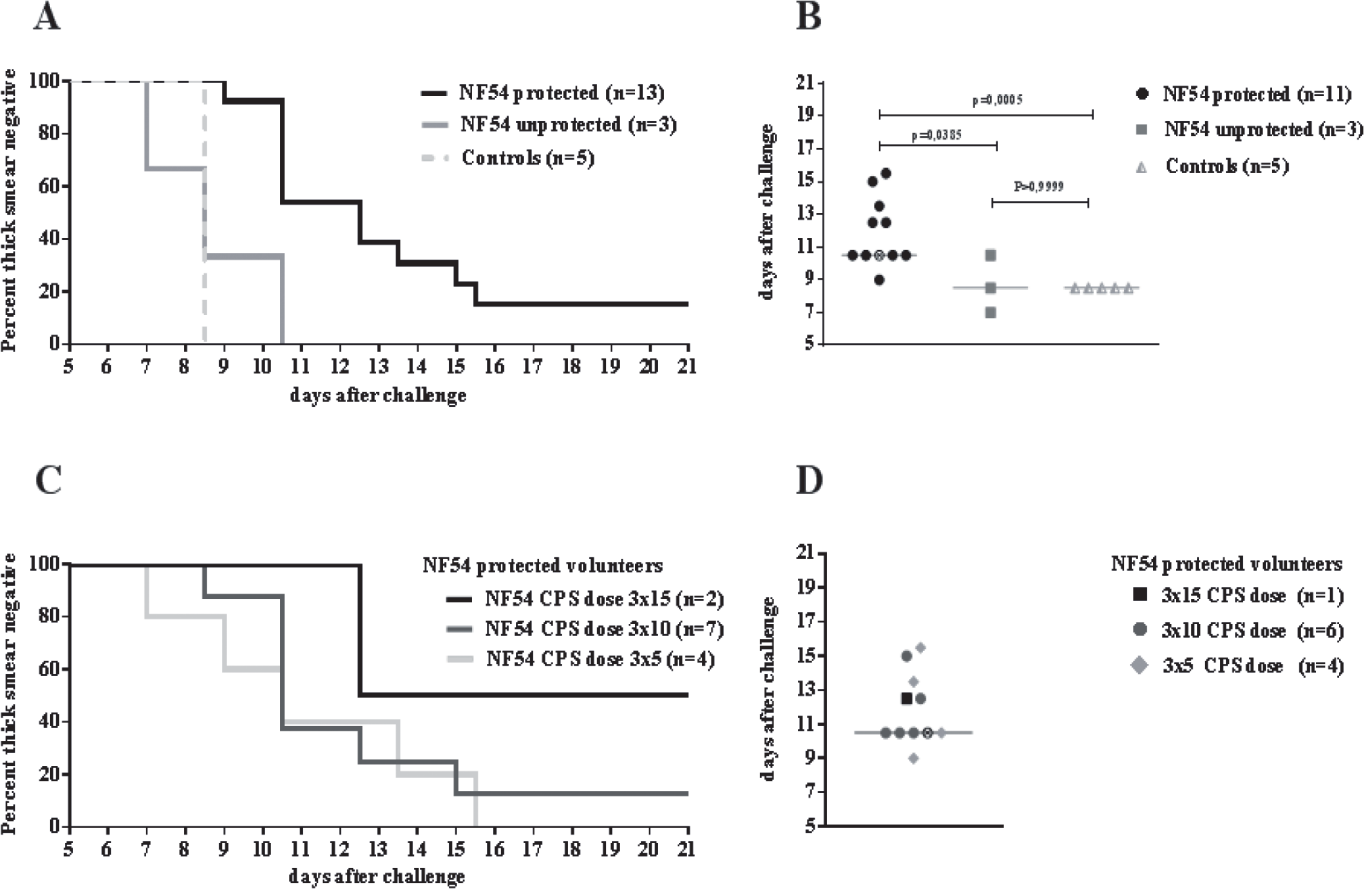
Protection and prepatent period after heterologous NF135.C10 challenge. **Left panels:** Kaplan-Meier curves showing percentage of thick smear negative volunteers after NF135.C10 challenge according to previous NF54 protection status (A) and NF54 CPS-immunization dose (C). **Right panels:** The corresponding distribution of prepatent period of thick smear positive volunteers is shown in dot plots according to NF54 protection status (B) and NF54 CPS-immunization dose (D). Lines represent medians. ⨂ = Volunteer presumptively treated after NF54 challenge and considered NF54 protected.

Sterile heterologous protection against NF135.C10 was complete in 15% (2/13) of NF54 protected volunteers **(Fig 2A)**. Patency was significantly delayed in the other 11 volunteers, indicative of partial protection (median prepatent period determined by thick smear was 10.5 days [range 9.0–15.5] versus 8.5 days [range 8.5–8.5] in controls; p = 0.0005 (**Table 1, Fig 2B**). Seven out of 11 partially protected subjects showed a delay in patency by qPCR of at least 48 hours, and thus more than one *P*. *falciparum* multiplication cycle.

**Table 1:** Protection against NF135.C10 challenge after NF54 CPS-immunization.

	NF135.C10	NF135.C10	
	Protected (n)	TS+ (n)	Prepatent period^a^
**NF54 protected**			
**3x15**	1	1	12.5
**3x10**	1	6	10.5 (10.5–15.0)
**3x5**	0	4	12.0 (9.0–15.5)
**all**	2	11	10.5 (9.0–15.5)***
**NF54 unprotected**			
**3x15**	0	0	
**3x10**	0	1	8.5
**3x5**	0	2	8.8 (7.0–10.5)
**all**	0	3	8.5 (7.0–10.5)
**Malaria-naive controls**	0	5	8.5 (8.5–8.5)

Sixteen previously CPS-immunized and challenged with *P*. *falciparum* NF54 volunteers in a CPS dose de-escalation and challenge trial were re-challenged with clone NF135.C10.

^a^Presented as median (range). N: number of volunteers. TS: Thick smear

***: p = 0.0005 compared to controls

**Fig 3:**
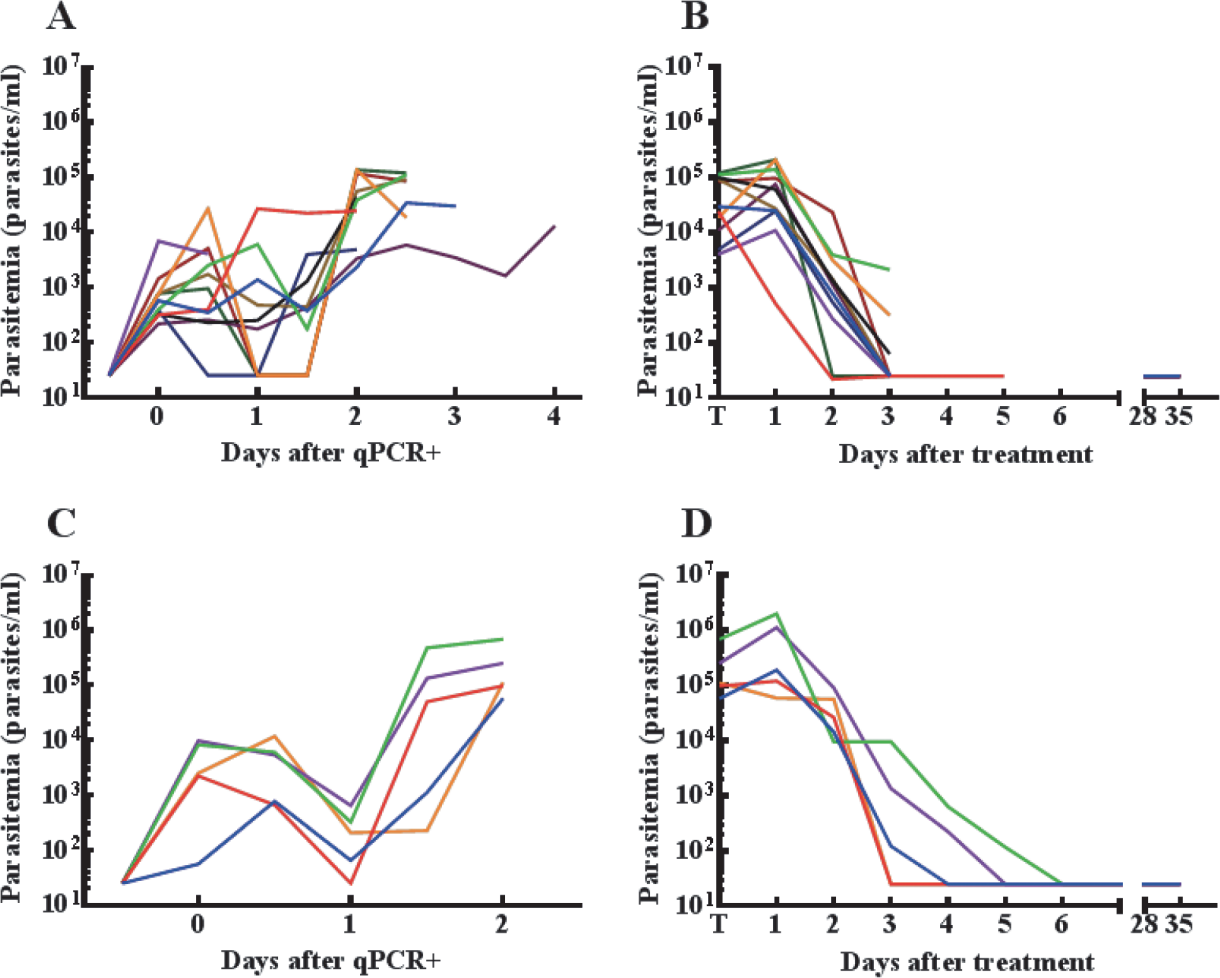
Parasitemia before and after treatment. Parasitemia measured by qPCR up until initiation of treatment (**A** and **C**) and from treatment onwards (**B** and **D**) in previously NF54 protected volunteers **(A** and **B)** and controls **(C** and **D)**. Each line represents an individual subject with the same colour before and after treatment. Values shown as 25 Pf/ml were negative (i.e. half the detection limit of the qPCR: 50 parasites/ml).

The 3 volunteers previously not protected against NF54 were neither protected against NF135.C10 (**Table 1, Fig 1**). The prepatent period by thick smear did not differ significantly between NF54 CPS-immunization dose groups (**Fig 2C and 2D**). Parasitemia at time of treatment was higher in controls compared to CPS immunized (p = 0.047; **Fig 3**).

### Adverse events

We next analysed adverse events in relation to the day of treatment to determine any early blood stage immune recognition to the parasite reflected in AE. Adverse events experienced by volunteers represent clinical manifestations of a malaria infection and can be possibly and probably related (both solicited and unsolicited) to the infection.

**Fig 4:**
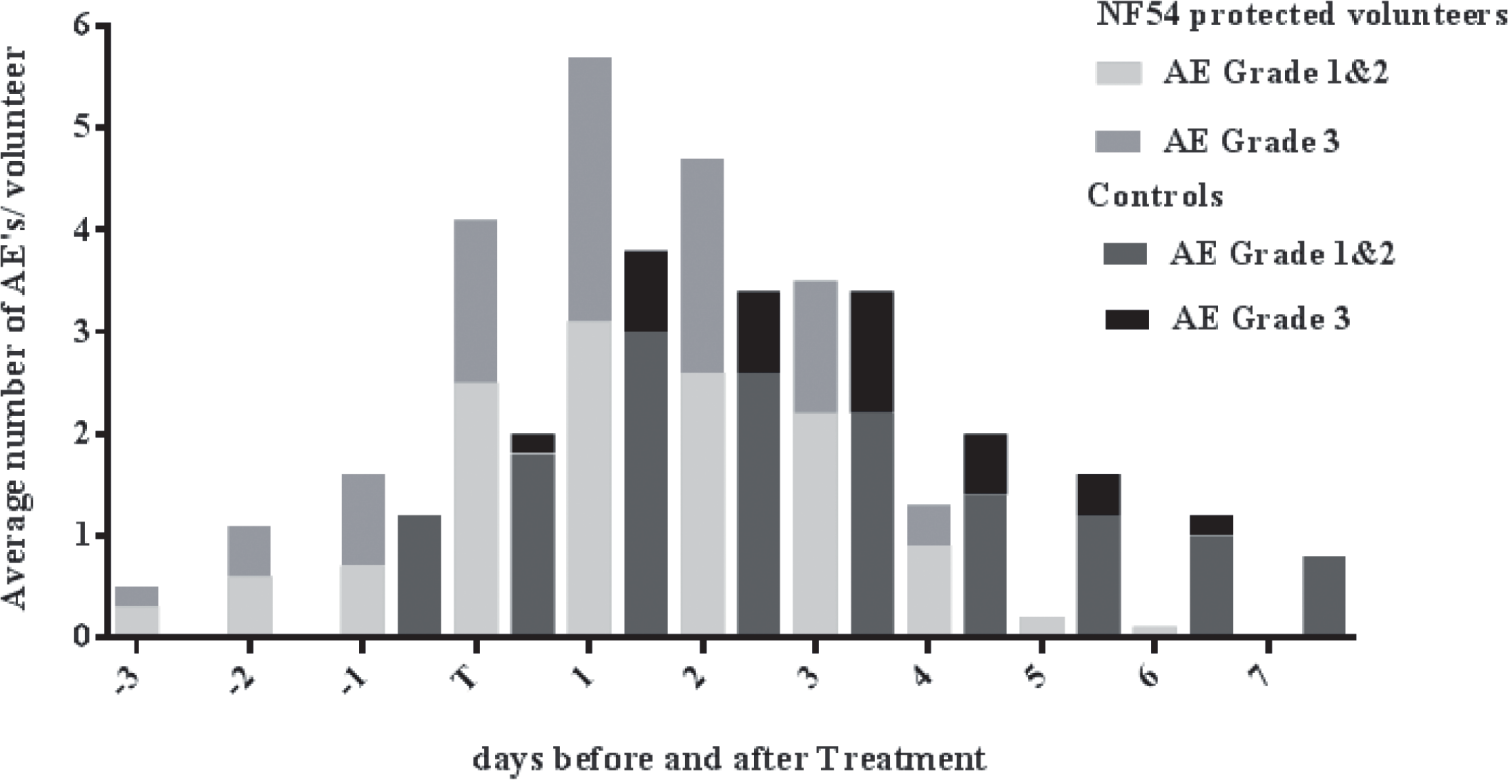
Adverse events before and after initiation of treatment. Average number of possibly and probably related (both solicited and unsolicited) AE per previously NF54 protected or control volunteer in relation to the time of positive thick smear (day of treatment). Time points are plotted towards day of treatment, depicted as ‘T’, from 3 days before until 7 days after start of treatment.

All volunteers reported possibly or probably related AE after challenge. Partially protected volunteers and controls showed a peak of AE on the first day after start of treatment (**Fig 4)**. Fourteen volunteers experienced related grade 3 AE, which were more frequently reported in partially protected than in control volunteers (8/10 versus 2/5 respectively). There were no serious AE. In partially protected volunteers, delayed patency concurred with earlier onset of AE in relation to detection of parasites by thick smear. While control volunteers did not experience any AE up until one day before detection of parasites by thick smear, partially protected volunteers experienced AE as early as three days before initiation of treatment.

All controls and one partially protected volunteer showed persisting fever (maximum 39.0°C) and/or mild to moderate complaints in the evening of day 3 after start of treatment. Resolution of the AE took longer (up to 7 days) in controls compared to partially protected volunteers, and to historical controls [11]. Additional thick smears performed in these volunteers on day 4, 5 and 6 after start of treatment were negative. All volunteers recovered fully without requiring additional antimalarial treatment.

### Safety parameters

Hs troponin T concentrations remained within normal range (<0.03 μg/L) in all volunteers. LDH was elevated in ten volunteers after initiation of treatment (median maximum value 242 U/L, range 182–718 U/L) and returned within normal range (0–248 U/L) during follow-up. D-dimer levels were elevated in all volunteers (median maximum value 1748 ng/ml, range 524 –<5000 ng/ml) and returned within normal range (0–220 ng/ml) during follow-up. The number of platelets decreased below lower reference value (150x10^9^/L) in 13 volunteers (median lowest value 127x10^9^/L, range 51–275x10^9^/L) without apparent clinical manifestations of bleeding or thrombotic complications. Safety parameters returned within normal range in all volunteers after treatment.

## Discussion

Our principle finding is that protection against a heterologous challenge infection with NF135.C10 is present in NF54 CPS-immunized and protected volunteers challenged more than one year before. Heterologous protection against NF135.C10 was complete in 15% (2/13) of volunteers while there was a delayed patency of more than 48 hours in 54% (7/13) of subjects. Taking into account a mean multiplication factor of 11.1 [11] and the presumed absence of functional blood stage immunity at this low parasitemia [3], this delay indicates that liver parasite load was reduced by approximately 91%. In three out of these seven volunteers a delay of more than two or three cycles was observed, indicating an estimated reduction of >99%. Three volunteers with no protection in the earlier homologous NF54 challenge study were also fully susceptible to NF135.C10.

Previous CPS studies showed that protection is mediated by immunity against pre-erythrocytic stages rather than asexual blood stages [3]. NF135.C10 originates from Cambodia, while NF54, isolated near Schiphol Amsterdam airport, likely originates from West Africa [9]. Both isolates show distinct differences in genes encoding three well-established antigens (MSP-1, MSP-2 and GLURP) as well as in the rif repetitive elements [9]. The target antigens of CPS-mediated protection remain to be elucidated in further studies including possible differences in antigen-specific responses to NF54 and NF135.C10.

Heterologous protection was incomplete in the majority of NF135.C10 re-challenged volunteers demonstrated by a delayed patency compared to controls. Apart from the genetic/antigenic variation between NF135.C10 and NF54, and thus insufficient breadth of the induced immune response, this incomplete heterologous protection may relate to a number of alternative explanations: i) Waning immunity: the heterologous challenge was performed at 14 months, rather than the usual 2 to 5 months post CPS-immunization; ii) Suboptimal sporozoite immunization dose received by the majority (14/16) of volunteers, indicating an antigen threshold for complete protection [8]. The minimally required immunization dose may increase for longevity of homologous protection and may be even higher for (long-lasting) heterologous protection. This trial was not powered to detect any dose-response relationships, but the two fully protected volunteers had indeed been immunized with the medium and high dose. iii) A possible difference between NF54 and NF135C.10 in sporozoite infectivity for liver cell invasion and/or maturation. This is supported by the higher first peak of NF135.C10 parasitemia was higher compared to historical NF54 controls (2871 *Pf*/ml versus 456 *Pf*/ml respectively [11].

In partially protected volunteers, delayed patency concurred with earlier onset of AEs. This might be due to the longer time-frame before parasitemia reaches the thick smear detection limit. Alternatively, early immune recognition of blood stage parasites by the host may result in an increased inflammatory response and subsequent increase in AEs. A comparable effect was observed in a previous trial, where CPS-immunized subjects who received a blood-stage challenge developed inflammatory markers and fever earlier than naïve controls [3].

Compared to partially protected volunteers, control volunteers showed prolonged AEs after treatment. This continuation of AEs until day 7 after treatment has not been observed in previous CHMI trials with either strain NF54 or NF135.C10, neither in the CPS studies nor in RAS studies [5]. Whether this represents an incidental finding or strain-specific characteristics needs to be investigated in future trials.

In conclusion, NF54 CPS-immunization induces heterologous protection against the geographically and genetically distinct *P*. *falciparum* NF135.C10 clone. Increasing the immunization dose, altering the immunizing strain, or even immunization with a combination of strains may further improve protection. These results and further optimization of CPS-immunization regimens will prove highly valuable for the clinical development of whole sporozoite vaccines.

## Supporting Information

S1 Checklist (PDF)

S1 Protocol (PDF)

S1 Flow Diagram (DOC)

S1 Methods (DOCX)
