# Supplementary material for: Heterologous Protection against Malaria after Immunization with Plasmodium falciparum Sporozoites
Source: PLoS One. 2015 May 1;10(5):e0124243. doi: 10.1371/journal.pone.0124243 (PMC4416703; doi:10.1371/journal.pone.0124243)
Supplement: S1 Protocol — (PDF) [file pone.0124243.s002.pdf]

CLINICAL TRIAL PROTOCOL

Re-exposure of previously immunized and challenged human volunteers to a heterologous strain of *P. falciparum* sporozoites

Version 3.0, 22 June 2012

TIP4

PROTOCOL TITLE Re-exposure of previously immunized and challenged human volunteers to a heterologous strain of *P. falciparum* sporozoites

**Protocol ID**	**NL39414.0000.12**
**Short title**	**TIP4**
**Version**	**3.0**
**Date**	**22 June 2012**
**Coordinating investigator**	**Remko Schats, MD LUMC**<br>Tel +31 71 526 2613<br>Fax: +31 71 526 6758<br>e-mail: r.schats@lumc.nl
**Principal investigator**	**Leo G. Visser, MD PhD LUMC**<br>Tel: +31 71 526 2613<br>Fax: +31 71 526 6758<br>e-mail: l.g.visser@lumc.nl
**Clinical investigators**	**Else M. Bijker, MD RUNMC**<br>Tel +31 24 3619515<br>Fax :+31 24 3614666<br>e-mail: e.bijker@ncmls.ru.nl<br>**Guido Bastiaens, MD RUNMC**<br>Tel +31 24 3619515<br>Fax :+31 24 3614666<br>e-mail: G.Bastiaens@ncmls.ru.nl
**Sponsor:**	*Radboud University Nijmegen Medical Centre*<br>*Department of Medical Microbiology*<br>**Prof. Robert W. Sauerwein, MD PhD**<br>Tel : +31 24 3610577<br>Fax :+31 24 3614666<br>e-mail: R.Sauerwein@mmb.umcn.nl
**Biological Evaluator:**	**Rob Hermsen, PhD RUNMC**<br>Tel : +31 24 3613663<br>Fax :+31 24 3614666<br>email : R.Hermsen@ncmls.ru.nl
**Grant**	**Top Institute Pharma**
**Independent physician(s)**	**Frank P. Kroon, MD PhD LUMC**<br>Tel: +31 71 526 2620<br>Fax: +31 71 526 6758<br>e-mail: F.P.Kroon@lumc.nl
**Safety monitor**	**Mark G.J. de Boer, PhD LUMC**<br>Tel: +31 71 526 5475<br>Fax: +31 71 526 6758<br>e-mail: M.G.J.de_Boer@lumc.nl
**Laboratory sites**	*Department of Parasitology*

	Department of Medical Microbiology<br>**Lisette van Lieshout, PhD LUMC**<br>Tel: +31 71 5265062<br>e-mail: E.A.van_Lieshout@lumc.nl<br>**Jaco J. Verweij, PhD LUMC**<br>Tel: +31 71 5265062<br>e-mail: J.J.Verweij@lumc.nl
**Pharmacy**	*LUMC Pharmacy*<br>Head pharmacist: **Prof. dr. HJ Guchelaar**<br>Trial assistent: **C.M. van der Hulst**<br>e-mail: c.m.van_der_hulst@lumc.nl<br>e-mail: trials@lumc.nl

PROTOCOL SIGNATURE SHEET

		Signature	Date
**Principal Investigator**	Leo G. Visser, MD PhD		
**Coordinating Investigator**	Remko Schats, MD		
**Sponsor**	Prof. Robert Sauerwein, MD PhD		

TABLE OF CONTENTS

LIST OF ABBREVIATIONS AND RELEVANT DEFINITIONS......7
SUMMARY......8
1. INTRODUCTION AND RATIONALE......10
1.1 Introduction......10
1.2 Rationale......11
1.3 Controlled Human Malaria Infections......12
1.4 Clinical Experience......12
1.5 Safety......13
2. OBJECTIVES......15
3. STUDY DESIGN......15
4. STUDY POPULATION......17
4.1 Population......17
4.2 Inclusion criteria......17
4.3 Exclusion criteria......18
4.4 Sample size calculation......19
5. TREATMENT OF SUBJECTS......21
5.1 Investigational product/treatment......21
5.2 Use of co-intervention......21
5.3 Escape medication......21
6. METHODS......21
6.1 Study parameters/endpoints......21
6.1.1 Main study parameter/endpoint......21
6.1.2 Secondary study parameters/endpoints......21
6.1.3 Exploratory study......22
6.2 Randomisation, blinding and treatment allocation......23
6.3 Study procedures......23
6.3.1 Mosquito challenge......24
6.3.2 Treatment with Malarone®......24
6.3.3 Mosquito preparation......25
6.3.4 Blood sampling......25
6.3.5 Case report forms and data collection......26
6.3.6 Flow chart trial procedures......27
6.4 Withdrawal of individual subjects......29
6.5 Follow-up of subjects withdrawn from treatment......29
6.6 Premature termination of the study......29
7. SAFETY REPORTING......29
7.1 Section 10 WMO event......29
7.2 Adverse and serious adverse events......30
7.2.1 Adverse events......30
7.2.2 Serious adverse events......30
7.2.3 Adverse Event Data Collection......30
7.2.4 Assessment of causality......31
7.2.5 Follow-up of adverse events......32
7.3 Local Safety Monitor and Data Safety Monitoring Board (DSMB)......32
7.3.1 Review of Safety Data by the Safety Monitor and DSMB......32

8. STATISTICAL ANALYSIS 34
9. ETHICAL CONSIDERATIONS 35
9.1 Regulation statement 35
9.2 Recruitment and consent 35
9.3 Benefits and risks assessment, group relatedness 35
9.3.1 Ethical aspects concerning the production of *P. falciparum* infected mosquitoes.. 35
9.3.2 Ethical aspects concerning the use of human volunteers 36
9.4 Compensation for injury 36
9.5 Incentives 36
10. ADMINISTRATIVE ASPECTS AND PUBLICATION 36
10.1 Handling and storage of data and documents 36
10.2 Amendments 37
10.3 Annual progress report 37
10.4 End of study report 37
10.5 Public disclosure and publication policy 37
10.6 List of Publication and Authorship 37
11. REFERENCES 38

LIST OF ABBREVIATIONS AND RELEVANT DEFINITIONS

**ABR**	**ABR form, General Assessment and Registration form, is the application form that is required for submission to the accredited Ethics Committee (In Dutch, ABR = Algemene Beoordeling en Registratie)**
**AE**	**Adverse Event**
**AR**	**Adverse Reaction**
**CA**	**Competent Authority**
**CCMO**	**Central Committee on Research Involving Human Subjects; in Dutch: Centrale Commissie Mensgebonden Onderzoek**
**CHMI**	**Controlled Human Malaria Infection**
**CV**	**Curriculum Vitae**
**DSMB**	**Data Safety Monitoring Board**
**EU**	**European Union**
**EudraCT**	**European drug regulatory affairs Clinical Trials**
**GCP**	**Good Clinical Practice**
**IB**	**Investigator's Brochure**
**IC**	**Informed Consent**
**IMP**	**Investigational Medicinal Product**
**IMPD**	**Investigational Medicinal Product Dossier**
**METC**	**Medical Research Ethics Committee (MREC); (in Dutch: Medisch Ethische Toetsing Commissie (METC))**
**Pf**	**Plasmodium falciparum**
**(S)AE**	**(Serious) Adverse Event**
**SPC**	**Summary of Product Characteristics (in Dutch: officiële productinfomatie IB1-tekst)**
**Sponsor**	**The sponsor is the party that commissions the organisation or performance of the research, for example a pharmaceutical company, Academical hospital, scientific organisation or investigator. A party that provides funding for a study but does not commission it is not regarded as the sponsor, but referred to as a subsidising party.**
**SUSAR**	**Suspected Unexpected Serious Adverse Reaction**
**Wbp**	**Personal Data Protection Act (in Dutch: Wet Bescherming Persoonsgegevens)**
**WMO**	**Medical Research Involving Human Subjects Act (in Dutch: Wet Medisch-wetenschappelijk Onderzoek met mensen)**

SUMMARY

Rationale:

Malaria is one of the major infectious diseases in the world with a tremendous impact on quality of life, significantly contributing to the ongoing poverty in endemic countries. It causes approximately 655.000 deaths per year, the majority of which are children under the age of five. The malaria parasite enters the human body through the skin, by the bite of an infected mosquito. Subsequently, it invades the liver, develops and multiplies inside hepatocytes. After approximately one week, the hepatocytes burst open and parasites are released in the blood stream, leading to the clinical phase of the disease.

As a unique opportunity to study malaria immunology and efficacy of immunisation strategies, a protocol has been developed to conduct controlled human malaria infections (CHMIs). CHMIs are studies consisting of a small group of malaria-naïve volunteers who are infected through bites of infected (e.g. *Plasmodium falciparum*) laboratory-reared Anopheles mosquitoes. Although potentially serious or even lethal*, P. falciparum* malaria can be radically cured at the earliest stages of blood infection when risks of complications are virtually absent and symptoms are usually mild.
We have shown previously that healthy human volunteers can be protected from a *P. falciparum* challenge by immunization with sporozoites (by mosquito bites) under chloroquine prophylaxis (CPS immunization). Interestingly, sterile protection in nearly all human CPS immunized volunteers was achieved by a relatively low dose, i.e. a total of 45 infectious mosquito bites.

In the ZonMw1 study (NL33904.091.10) we challenged 24 volunteers after Chloroquine Profylaxis Sporozoites (CPS) immunization with 45, 30 or 15 infected mosquito-bites respectively. The availability of this immunized cohort opens the unique opportunity to determine protection to a heterologous challenge for both of the protected and unprotected volunteers as the previous challenge infection might have served as immunological boost to the unprotected volunteers.
In the current observational, proof of principle study, we aim to investigate the protection on an individual basis of these previously immunized and challenged volunteers against a heterologous *P. falciparum* NF135.C10 challenge.

Objectives:

Primary Objective: To determine protection against a heterologous *P. falciparum* re-challenge after Chloroquine Prophylaxis Sporozoites (CPS) immunization and challenge.

Secondary Objectives:

- To study and compare the development of parasitemia after challenge between protected and unprotected individuals
- To study and compare signs and symptoms after challenge between protected and unprotected individuals
- To analyse and compare the immune responses between protected and unprotected individuals

Study design: The study is a single centre open label clinical trial. Laboratory personnel will be

blinded, volunteers and the investigators will both not be blinded.

Study population:
A maximum of 25 healthy volunteers, aged 18 to 35 years, male and female, will participate in the study.
All immunized and challenged volunteers from the ZonMw1 study will be asked to participate in this follow-up study, a maximum of 20 and a minimum of 10 volunteers. In addition, five malaria-naïve healthy volunteers (age ≥ 18 and ≤ 35 years, males or females) will be recruited to form a control group.

Study procedure:
All volunteers will be exposed to five *P. falciparum* NF135.C10 infected mosquito-bites. Following exposure, volunteers are required to stay in the study hotel from day 5 post infection until 3 days after start of treatment. All volunteers will be treated with a curative regimen of Malarone® (atovaquon/proguanil); once daily 4 tablets of 250/100mg during three days. Criteria for treatment are as follows:

- Positive thick smear is on regular check-up
- Symptoms of malaria-infection and positive thick smear
- By decision of study doctor or the safety monitor
- On request of the volunteer
- On day 21, if the volunteer has remained thick smear negative

Study parameters/endpoints:
Primary endpoint:
Duration of prepatent period after challenge infection as measured by microscopy

Secondary endpoints:
- Parasitemia and kinetics of parasitemia as measured by PCR
- Frequency of signs or symptoms in study groups
- Immune responses between study groups

Nature and extent of the burden and risks associated with participation, benefit and group relatedness:
Benefits: No benefit can be claimed for any of the volunteers. Even though previously immunized and challenged volunteers (ZonMw1) might be protected to a different strain of *P. falciparum* in this study, these effects may not apply to field situations. Therefore, volunteers will be advised to take adequate malaria prophylaxis and preventative measurements (e.g. bednet, deet etc) when travelling to malaria endemic areas in the future.
Risks: Risks for volunteers are related to exposure to (early) *P. falciparum* malaria infection and side-effects of Malarone® treatment.
Burden: This study involves a challenge infection in which volunteers receive mosquito bites on day 0, and have to make visits to the trial centre during a period of 140 days afterwards, starting on day 5 after challenge.

After challenge there will be a period (35 days) of intense clinical monitoring with frequent site visits and blood examinations. The first two days involve once daily visits. The next nine days will involve twice daily visits, the six days afterwards once a day. After start of Malarone treatment, volunteers visit the trial centre twice a day. As it is unpredictable if and/or when subjects will develop a positive thick blood smear after challenge infection, it is impossible to state the exact number of site visits and blood examinations. However, the maximum number (in case a subject does not develop a positive blood smear) of site visits will be 39 (34 blood examinations) with a maximum amount of collected blood of 500 mL. In addition periodical physical examinations will be performed and the subject is asked to complete a diary.

1. INTRODUCTION AND RATIONALE

1.1 Introduction

Malaria is a common and serious infectious disease, endemically and epidemically present throughout low and medium income countries (LOMIC). It is a parasitic infection transmitted by mosquitoes. In humans malaria is caused by five subspecies of *Plasmodium* protozoa with *P. falciparum* as most serious and detrimental

Malaria parasites (sporozoite stage) are injected in the skin by a female Anopheles sp. mosquito. From there they travel via the bloodstream to the liver, where they develop and multiply in liver cells for approximately 7 days before entering the blood stream again (merozoite stage) and invading red blood cells for further reproduction. Clinical malaria is caused by the rupture of parasitized erythrocytes, and subsequent release of large amounts of asexual stages of Plasmodium in the blood.

Malaria is a public health problem in over 106 countries worldwide, and affects approximately 40% of the world population, with over 3.3 billion people at risk. It has been estimated that the incidence of malaria in the world may be in the order of 216 million clinical cases each year (WHO Malaria report 2011). People living in sub-Saharan African countries account for more than 90% of these malaria cases. Malaria mortality was estimated at 655.000 deaths worldwide in 2010. Most malaria deaths occur among children below the age of five in Africa, especially in remote rural areas with poor access to health services. Other high-risk groups include women during pregnancy, refugees and non- immune travelers. The epidemiology of malaria has been changing over recent years due to a combination of factors including increasing resistance of malarial parasites to chemotherapy and increasing insecticide resistance of the *Anopheles* mosquito vectors, ecological and climate changes and increased international travel to malaria-endemic areas.

There are several reasons why malaria continues to be one of the greatest public health challenges. Apart from political and social factors in a country that often affects effective implementation of national malaria control programmes; other key factors are inconsistent access and usage of available tools. Tools include availability of adequate, quality controlled, drugs and consistent usage of preventative measures such as long lasting insecticide-treated bed nets (LLITN) and effective pyrethroid class of insecticides. The availability of an efficacious malaria vaccine would certainly be a major additional tool to overcome the shortcomings of current control strategies.

1.2 Rationale

Both natural and experimental exposure to malaria parasites can lead to development of protective immunity, providing a foothold for the development of a vaccine (Doolan 2009; Beeson 2008; Pombo 2002; Hoffman 2002; Roestenberg 2009). The clinical development of a malaria vaccine has been a continuous effort over the past half century (Epstein 2007) following the traditional vaccine development approach. Different formulations of a number of antigens and/or adjuvants have been tested in Phase I trials but only about a dozen candidates have been evaluated in Phase II clinical field trials.
(http://www.who.int/vaccine_research/links/Rainbow/en/index.html).
No vaccine has worked well and most candidates have failed completely. The best vaccine to date, RTS,S, protects up to 46% of children, reduces clinical severity by 55% during 12 months only (Abdulla 2008; Breman 2009, Agnandji 2011). Although a milestone in itself and potentially an additional tool in the combat against malaria, it is clear that better vaccine efficacies are required (Targett 2008).

A more effective alternative is immunization with sporozoites, attenuated by irradiation, which has been shown to induce strong protective immunity in rodents and in humans (Collins 1972; Hoffman 2002). Irradiation disrupts gene expression of sporozoites, which remain capable of hepatocyte invasion but do not complete liver-stage maturation and do not progress to the pathogenic blood stage (Silvie 2002). However, induction of protective immunity in humans with this model requires a minimum of 1000 bites by irradiated mosquitoes during five or more immunization sessions, making practical application impossible. A technology platform to administer radiation attenuated sporozoites by syringe rather than mosquito bites is in progress but not yet available (Hoffman 2010).

A highly efficient induction of sterile protection against malaria can be achieved in rodents and humans by inoculation of intact sporozoites while concomitantly treating with chloroquine, a drug that kills parasites in the asexual blood stage but not in the pre-erythrocytic liver stage (CPS immunization; Belnoue 2004, Roestenberg 2009: see section K4a). Interestingly sterile protection in nearly 100% of the human CPS immunized volunteers is achieved by a relatively low dose, i.e. a total of 45 infectious mosquito bites, strikingly 20-fold more potent than the 1000 bites needed in the model using irradiated mosquitoes. This dose is also much lower than in malaria endemic areas where many years of natural exposure to infected mosquitoes are needed to achieve (clinical) protection against malaria. Furthermore, protection has been shown to last up to 28 months (Roestenberg 2011), which is much longer than generally recorded after natural exposure and longer than the current RTS,S vaccine.
One of the hypotheses raised to explain the highly efficient protective immunity induced by CPS immunization, is that the challenge infection in these studies was performed with a homologous *P. falciparum* strain. However, in the field there is a huge variety of strains and antigenic repertoire. It is therefore essential that a vaccine protects against multiple strains.

In the distant past, heterologous challenge infections after immunizations have been performed in only 5 volunteers with minimal clinical and parasitological reporting (Hoffman 2002, Rieckmann 1974). The *P. falciparum* strain that was used for CPS immunization in trial was the NF54 strain, which is known to easily produce sexual stage parasites that are essential for the production of infectious mosquitoes. NF135.C10 was a fresh *Pf* isolate obtained from a Dutch traveller to Cambodia. Parasites were adapted to continuous culture and subsequently cloned as

NF135.C10. This clone has been consistently producing gametocytes and generating substantial numbers of sporozoites in laboratory reared *Anopheles* mosquitoes. This clone was tested in a controlled human malaria infection showing similar clinical presentation and development of parasitaemia as NF54 (Sauerwein, unpublished)

In the ZonMw1 study (NL33904.091.10), we challenged a total of 24 volunteers after CPS immunization of three groups with 45, 30 or 15 infected mosquito-bites respectively. Seventeen of 24 these volunteers were fully protected against a homologous challenge after CPS immunization while 7 volunteers developed parasitaemia with similar prepatent period as the control group. The availability of this immunized cohort opens the unique opportunity to determine protection to a heterologous challenge for both of the protected and unprotected volunteers as the previous challenge infection might have served as an immunological boost to the unprotected volunteers.
We therefore aim to investigate through an observational, proof of principle, study to assess the protection on an individual basis of all previously immunized and challenged volunteers against a heterologous *P. falciparum* NF135.C10 challenge.

1.3 Controlled Human Malaria Infections

Controlled human malaria infections are well accepted as a powerful tool for the evaluation of parasite development in humans. Parasite multiplication is the key parameter in such trials, which implies that parasitemia should be followed over a sufficiently lengthy period. For determination of both erythrocytic and pre-erythrocytic parasite multiplication, accurate measurement of parasitemia after exposure to infectious bites is absolutely essential. We have the experience and infrastructure to conduct controlled human malaria infections. We have also developed a very sensitive method of parasite detection by Real-time Quantitative PCR (RTQ-PCR) that will allow us to detect small differences in parasite density. Using these sensitive measurements of parasitemia, we have developed a statistical model of parasitemia after mosquito bite (Hermsen 2004). We will use our model to calculate numbers of infected liver cells and parasite multiplication factors in human volunteers over time.

1.4 Clinical Experience

There is a large clinical experience with infecting humans by the bite of *P. falciparum* sporozoite-infected mosquitoes. Since 1986 more than 1400 volunteers are challenged by the bites of mosquitoes fed on cultures of *P. falciparum* gametocytes to produce sporozoites (Chulay 1986). This has proved to be a reproducible, predictable and safe method of inducing a controlled *P. falciparum* infection. The results of such studies were summarized in 1997 (Church 1997), in 2007 (Epstein 2007) and in 2012 (Roestenberg 2012, in press).
Controlled human malaria infections have been conducted in the Centre for Clinical Malaria Research (CCMS) Nijmegen in 200 volunteers since its origin in 2001. Controlled human malaria infections have also been conducted in 45 volunteers in Leiden in collaboration with the Centre for Clinical Malaria Research (CCMS) Nijmegen since 2009. Standard operating procedures according to international standards are in place for both clinical and laboratory activities at both sites.

1.5 Safety

In February 2008 a cardiac SAE (CCMO08.1096/MA/14715) in a 20 year old female participating in LSA3/Alhydrogel (LSA-3 CMO-07/37; NL14715.000.06) malaria vaccine trial was reported to the CCMO. The findings have been published as a case report titled “Cardiac complication after controlled human malaria infection: case report”, A.E. Nieman et al, Malar. J. 2009 Dec 3;8(1):277 (See section K4b). The true nature and patho-physiological explanation of the event remain unclear. Following this event, recommendations of the European Malaria Vaccine Development Association and the CCMO to ensure maximal safety of participating human volunteers have been integrated in the controlled human malaria infection protocol:

1. Riamet® is no longer used as treatment for controlled human malaria infections. It is replaced by Malarone®.
2. Volunteers with 1st or 2nd degree relatives with cardiac events under the age of 50 will be excluded from participation.
3. The occurrence of the cardiac SAE is included as part of the volunteer information sheet.
4. Volunteers will be required to stay at very close distance to the LUMC to ensure maximal safety from day 5 after challenge, until treatment has been finished (maximum 20 days).
5. Negative urine toxicology screening test is added as inclusion criterion.
6. Monitoring of highly sensitive troponin T (hsTropT), D-dimer, lactate dehydrogenase (LDH), thrombocytes, ADAMTS13 and fragmentocytes.

Since above mentioned adaptations, five more controlled human malaria infection studies have been concluded in a total of 99 volunteers (NL30350.058.09, NL24193.091.09, NL31858.091.10, NL34273.091.10 and NL33904.091.10). No cardiovascular adverse events occurred in any of the volunteers. Monitoring of cardio-vascular markers showed the following results:

After challenge, fifty-seven out of sixty-two volunteers with a positive thick smear developed elevated d-dimer levels, twelve volunteers (also) had elevated d-dimer levels without a positive thick smear. Median d-dimer peak concentration was 2500 ng/ml (n=65), range 540-14600 ng/ml.

After immunization by infected mosquito-bites under chloroquine prophylaxis, two volunteers had an elevated d-dimer with a positive thick smear (maximum 9670 and 980 ng/ml), and twenty-four volunteers had an elevated d-dimer without a positive thick smear (median 1200 ng/ml, range 537-3950 ng/ml).

In all cases, d-dimer values normalized during follow-up.
ADAMTS13 and fragmentocytes were investigated in twenty-one cases after challenge-infection and in seventeen cases after immunization with infected mosquito bites under chloroquine prophylaxis. The number of fragmentocytes was always within the normal range. ADAMTS13 approached abnormal values (38%) in one volunteer, but normalized activity was noticed in the follow-up period. Average ADAMTS13 activity was 97% (n=38).

HS-Troponin T values were slightly elevated (0.047 µg/L) in one asymptomatic volunteer just after extremely intensive sports activity. The DSMB of this trial (ZonMw1, NL33904.091.10) concluded that this elevation in HS-Troponin T did not indicate cardiac damage, but was due to the sports activity and therefore this should not have consequences for the volunteer or the trial. Otherwise, no abnormal HS-Troponin T values occurred in any of the studies.

A serious adverse event occurred in a female volunteer in the TIP2 trial (NL31858.091.10). She reported chest pain the day after start of atovaquone/proguanil treatment for Pf parasitemia. Based on medical history, the chest pain when first seen was considered possibly consistent with angina pectoris. It resolved within one hour without treatment. She was admitted to the cardiac care unit for monitoring for 6.5 hours. The ECG showed a negative T-wave in V2, which had been absent at screening, but could have been caused by positioning of the electrodes. Subsequent EKG's were comparable to baseline, with a negative T in V1 only. Highly sensitive troponin T (detection limit 0.003 µg per litre) did not show any changes at the time of chest pain, six hours later, 17 hours later, daily for three days and at trial day 28 and 35. The trial was put on hold pending discussion with the Sponsor, the Safety Monitoring Committee (SMC), and the U.S. F.D.A. Since there was no more chest pain or changes in the EKG during the subsequent three days, the SMC considered the event not to be cardiac, and recommended the trial be resumed according to protocol within three days of the event.

A serious adverse event occurred in a male volunteer in the ZonMw1 trial (NL33904.091.10). Elevated creatine phosphokinase, ASAT, LDH and ALAT levels were measured 10 days after challenge infection. History revealed weightlifting and two subsequent visits to a sauna. Therefore, enzyme elevations were classified as exertional rhabdomyolysis, aggravated by two sauna visits. Since thick smears and PCR were negative for Plasmodium falciparum, the DSMB considered the event not related to the trial.

2. OBJECTIVES

Primary Objective: To determine protection against a heterologous *P. falciparum* re-challenge after Chloroquine Prophylaxis Sporozoites (CPS) immunization and challenge.

Secondary Objectives:

- To study and compare the development of parasitemia after challenge between protected and unprotected individuals
- To study and compare signs and symptoms after challenge between protected and unprotected individuals
- To analyse and compare the immune responses between protected and unprotected individuals

3. STUDY DESIGN

The study is a single centre open label clinical trial. Laboratory personnel will be blinded, volunteers and the investigators will both not be blinded.

A maximum of 25 volunteers will be divided into two groups as shown in Table 1. All volunteers will be challenged by the bites of 5 mosquitoes, infected with the *P. falciparum* NF135.C10 strain.

Table 1, study groups

**Group number**	**Challenge with infected mosquito-bites**
1. n=20; Immunized and challenged subjects ZonMw1	Yes
2. n=5; control group	Yes

A summary of the study schedule is shown in figure 1.

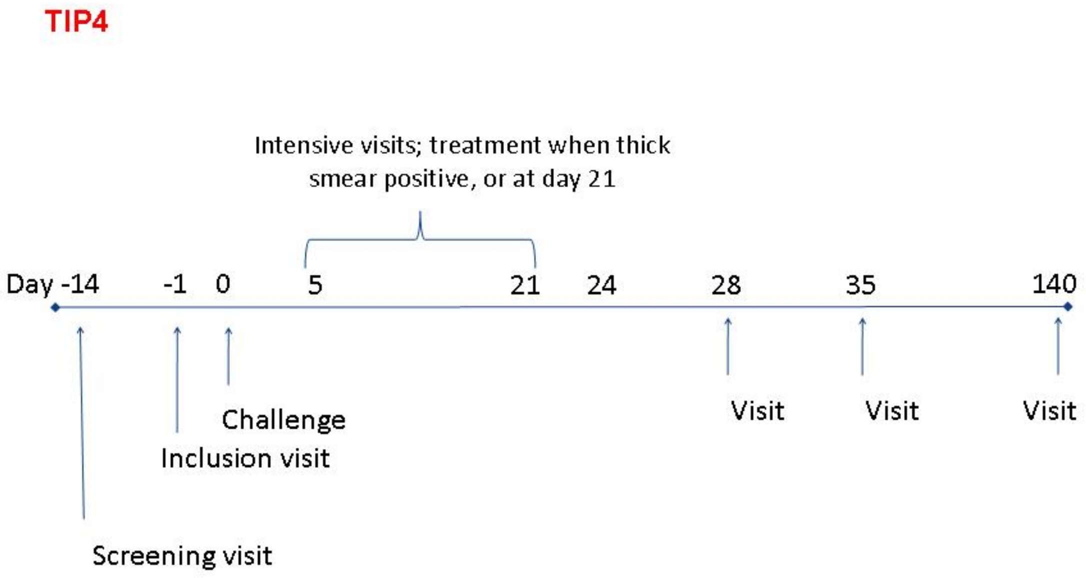
TIP4
Intensive visits; treatment when thick smear positive, or at day 21
Day -14
-1
0
5
21
24
28
35
140
Challenge
Inclusion visit
Screening visit
Visit
Visit
Visit

4. STUDY POPULATION

4.1 Population

A maximum of twenty previously immunized and challenged volunteers from the ZonMw1 study will be asked to participate in this study. In addition five healthy, malaria naïve volunteers will be recruited. This study will not commence without a minimum of 10 previously immunized volunteers from the ZonMw1 study and 5 volunteers for the control group.

Advertisements will be placed in prominent places on different University campuses and other public places as well as on the intranet of the institutions. Furthermore, a facebook-page showing the advertisement-text will be designed to inform people about the trial. This brief advertisement (section E3) will indicate a telephone number to call and an email address for contact to request further information. It will furthermore indicate a website which contains a form. A general, short questionnaire will be completed over the phone, over email or by the form on the website. When seemingly suitable, volunteers will be invited to an information meeting, in which the study is explained to them by the study investigator, and given the information sheet (section E1), the application form (section F1a) and the insurance text (section E4) to study them at home. During and after the meeting there will be ample room for questions. Volunteers willing to apply fill in the application form and are invited to come to the screening visit. At this screening visit, the informed consent form (section E2) will be signed, the questionnaire answers will be discussed and inclusion and exclusion criteria will be checked. Also, a letter for the general practitioner will be signed and sent after screening (section K6a). Again, there will be ample room for questions. The possibility of withdrawal from the study, at any time and without any declaration of the reason will be pointed out to the volunteers. If volunteers prove to be eligible, they will be invited to the next visit.

4.2 Inclusion criteria

1. Age ≥ 18 and ≤ 35 years healthy volunteers (males or females)
2. Good health based on history and clinical examination
3. Negative pregnancy test
4. Use of adequate contraception for females
5. Signing of the informed consent form, thereby demonstrating understanding of the meaning and procedures of the study
6. Agreement to inform the general practitioner and to sign a request to release medical information concerning contra-indications for participation in the study
7. Willingness to undergo a *Pf* controlled challenge through mosquito bites
8. Agreement to stay in a hotel room close to the trial centre during a part of the study (Day 5 after challenge till treatment is finished)
9. Reachable (24/7) by mobile phone during the whole study period
10. Available to attend all study visit
11. Agreement to refrain from blood donation to Sanquin or for other purposes, during the whole study period
12. Willingness to undergo HIV, hepatitis B and hepatitis C tests
13. Negative urine toxicology screening test at screening visit and the day before challenge
14. Willingness to take a curative regimen of Malarone®

4.3 Exclusion criteria

1. History of malaria (other than participation in ZonMw1 study) or residence in malaria endemic areas within the past six months
2. Plans to travel to malaria endemic areas during the study period
3. Plans to travel outside of the Netherlands during the challenge period
4. Previous participation in any malaria vaccine study and/or positive serology for *Pf (except ZonMw1 volunteers)*
5. Symptoms, physical signs and laboratory values suggestive of systemic disorders including renal, hepatic, cardiovascular, pulmonary, skin, immunodeficiency, psychiatric, and other conditions which could interfere with the interpretation of the study results or compromise the health of the volunteers
6. History of diabetes mellitus or cancer (except basal cell carcinoma of the skin)
7. History of arrhythmias or prolonged QT-interval
8. Positive family history in 1st and 2nd degree relatives for cardiac events < 50 years old
9. An estimated, ten year risk of fatal cardiovascular disease of ≥5%, as estimated by the Systematic Coronary Risk Evaluation (SCORE) system
10. Clinically significant abnormalities in electrocardiogram (ECG) at screening
11. Body Mass Index (BMI) below 18 or above 30 kg/m2
12. Any clinically significant deviation from the normal range in biochemistry or hematology blood tests or in urine analysis
13. Positive HIV, HBV or HCV tests
14. Participation in any other clinical study within 30 days prior to the onset of the study
15. Enrolment in any other clinical study during the study period
16. For women: being pregnant or lactating
17. Volunteers unable to give written informed consent
18. Volunteers unable to be closely followed for social, geographic or psychological reasons
19. History of drug or alcohol abuse interfering with normal social function
20. A history of treatment for psychiatric disease
21. A history of convulsions
23. Contra-indications to Malarone®, including hypersensitivity or treatment taken by the volunteer that interferes with Malarone®
24. The use of chronic immunosuppressive drugs, antibiotics, or other immune modifying drugs within three months of study onset (inhaled and topical corticosteroids and oral anti-histaminic are allowed) and during the study period
25. Any confirmed or suspected immunosuppressive or immunodeficient condition, including (functional) asplenia
26. Co-workers or trainees of the departments Infectious Diseases, Medical Microbiology or Parasitology of the Leiden University Medical Centre (LUMC) or Medical Microbiology, Parasitology, Radboud University Nijmegen (RUNMC)
27. A history of sickle cell anaemia, sickle cell trait, thalassaemia (or trait), G6PD deficiency

4.4 Sample size calculation

Our primary objective is to determine protection against a heterologous *P. falciparum* re-challenge after previous CPS immunized and challenged (ZonMw1) volunteers on an individual basis. We include a minimum of 10 and a maximum of 20 ZonMw1 volunteers in the current study.

A statistical model of parasitemia after mosquito infection has been developed, which is based on quantitative real-time PCR measurements of parasitaemia (figure 1) (Hermsen, C.C. et al, *Am.J.Trop.Med.Hyg.* 2004. 71: 196-201).

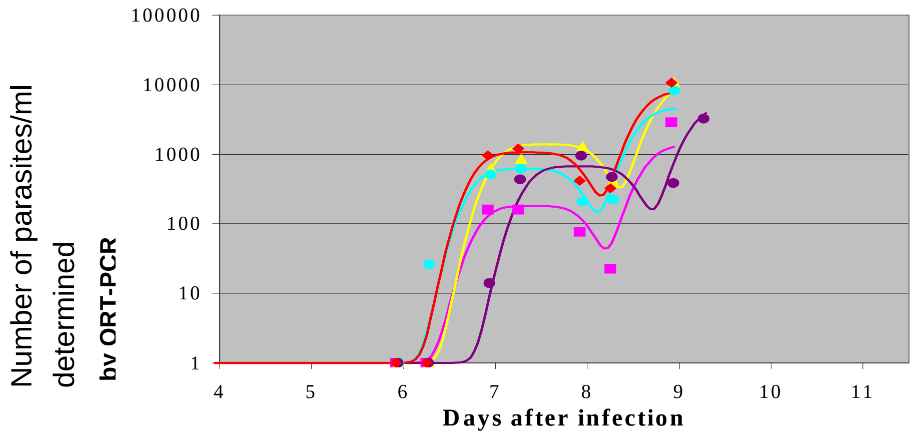

Figure 1: Observed and predicted parasite densities of 5 representatives out of 15 individuals experimentally infected with *Plasmodium falciparum*. Markers represent observed number of parasites per ml blood based on the quantitative real-time PCR results. Observations of one individual have the same colour. Individual observations were continued until a standard blood smear was found positive, after which immediate treatment was provided. The best fitting curve for each individual is represented in the same colour as the corresponding markers.

We used our model to simulate observations for non-protected and protected experimentally infected human volunteers over time (Hermsen et al, Am J Trop Med Hyg. 2004 Aug;71(2):196-201). Three sources of variation, as determined from previous testing of the model against data from 15 volunteers, were included in the simulations: (1) individual variation in level of infection (number of infected hepatocytes); (2) individual variation in timing of the first generation of ring forms after the hepatocyte stage; and (3) variation in measurement error. Previously estimated parameters for mean/variance of duration of presence/absence of ring forms and the multiplication factor between successive parasite cycles were pre-fixed. This quantitative analysis permits a detailed estimation of critical parameters in the parasite life cycle including duration of trophozoite maturation and multiplication of blood stages due to a remarkable consistent pattern of PCR positive parasitaemia before the blood slide becomes positive and treatment is required.

In this project, the main protective effects are expected at the level of the liver stage, but some protective mechanisms may operate on asexual blood forms. We made a power calculation for the minimal degree of asexual stage protection that could be shown with a group size of 5

volunteers. The effect of a 60% effective asexual stage protection was mimicked by a 60% reduction of the number of ring forms per hepatocyte and the multiplication factor. Figure 2 below gives a typical example of two groups of 10 individuals. In this example, parasite counts of both groups only partly overlap between 7 – 8 days after infection, and significantly differ >8 days after infection. To do power analysis, such simulations were repeated 100 times. From this we conclude that testing 60% effective asexual stage protection using 5 individuals per group and α = 5% will give significant results in more than 90% of the experiments). When using 10 individuals per group, this will be >95%.

5. TREATMENT OF SUBJECTS

5.1 Investigational product/treatment
There is no investigational product in this study.

5.2 Use of co-intervention
All volunteers will be treated with Malarone® (tablets containing 250 mg atovaquon and 100 mg of proguanil) as described in section 6.3.4.

5.3 Escape medication
Volunteers are advised to take tripelennamine crème for the local treatment of mosquito bites. Volunteers are advised to take paracetamol for complaints secondary to the mosquito challenge (fever, muscle aches, headache, etc.). Tripelennamine crème, paracetamol or any other symptomatic treatment will be supplied to the volunteers. The maximum dose of paracetamol is 4 grams a day.

6. METHODS

6.1 Study parameters/endpoints

6.1.1 Main study parameter/endpoint
- Duration of pre-patent period after challenge infection as measured by microscopy

Thick smear samples will be taken from a 3 ml EDTA vacutainer tube. Thick smears will be performed on all visits following challenge infection until treatment is finished.
Thick smears will be performed according to a standard operating procedure which is based on an internationally harmonized protocol for thick smears in CHMIs (Moorthy et al., WHO). In short, 15µl of whole blood will be distributed on standardized 3-well slides, providing an equal slide thickness for all smears. Slides are dried and coloured with Giemsa staining. Per slide, 200 fields will be read. Slides are considered positive if they contain 2 or more parasites per 200 fields. The sensitivity of the thick smear microscopy is 4 parasites per µl.

6.1.2 Secondary study parameters/endpoints
- Development of parasitemia as measured by PCR
- Frequency of signs or symptoms in study groups
- Immune responses between study groups

Samples for RTQ-PCR will be collected from the same 3 ml EDTA vacutainer tubes as the thick smear sample. RTQ-PCR will be performed according to standard procedure described in Hermsen et al. Mol, Biochem. Parasitol. 2001;118: 247-251. In short, RTQ-PCR will be performed on the multicopy 18S ribosomal RNA gene. All samples are spiked with murine white blood cells and a murine albumin gene PCR is used to determine efficacy of DNA isolation.
Samples for quantitative measurement of parasitemia will be prepared and stored at LUMC. Measurement by RTQ-PCR will be performed retrospectively at the department of Parasitology of the LUMC in collaboration with the Radboud University Nijmegen Medical Centre department of Medical Microbiology.

Signs and symptoms will be recorded at all visits, and whenever a trial volunteer reports signs or symptoms to the trial physician between visits. The following signs and symptoms will be solicited: fever, headache, malaise, fatigue, myalgia, arthralgia, nausea, vomiting, chills, diarrhoea, abdominal pain (Verhage 2005), chest pain, palpitations and shortness of breath. For more information on recording of adverse events please refer to section 7.2.

We aim to answer the following immunological questions:
a) Which cellular and humoral immune responses are associated with protection against a heterologous challenge infection?
b) Is there a set of biomarkers on transcriptional level that predict protective immunity against re-challenge with heterologous sporozoites?
c) How does *P. falciparum* infection modify innate and adaptive immune cell activation?

To address these questions, several immunological assays will be performed. These will include phenotyping of peripheral blood mononuclear cells (PBMC) by flow cytometry as well as a number of functional assays to determine antigen-specific responses. T-cell responses will be evaluated using stimulation assays in which PBMCs of volunteers will be in vitro stimulated with parasite stimuli and antigens. Readouts will include flow cytometry and ELISpot. B-cell functional assays will be performed both on plasma samples and PBMCs using ELISpot, ELISA and protein microarray. PBMC's for these assays will be collected at day C -1 (pre-challenge), day of treatment, three days after treatment (T+3), day C+35 and day C+140 (follow-up) by venapuncture and collected in CPT tubes..

Additionally, we will determine immune responses at sequential time points during challenge on the transcriptional level using mRNA expression analysis by dual-color Reverse-Transcriptase Multiplex Ligation-dependent Probe Amplification (dcRT-MLPA).

Samples for dcRT-MLPA will be collected at: day -1 (pre-challenge), day C+5, day C+6, day C+9, day of treatment (T) and day C+35 (follow-up). At each of given time points 1 ml of leftover blood will be used from the haematology EDTA tube (blood used for safety purposes) to perform a lymphocyte subset analysis. These data will be used to improve the interpretation of the dcRT-MLPA data.

6.1.3 Exploratory study endpoint

- Metabolomic responses between study groups
- Transcriptome following challenge between groups

Metabolomics is the quantitative measurement of the global and dynamic metabolic processes of the response of organisms to physical changes, for example infectious stimuli like malaria (Nicholson 2008). We aim to perform an exploratory study measuring differences in metabolic profile in between protected and unprotected volunteers for a *P. falciparum* heterologous strain.

Metabolic endpoints will be measured at same time points during the study as immunological dcRT-MLPA endpoints: day -1 (pre-challenge), day 5, day 6, day 9, day of treatment (T) and day 35 (follow-up).

Leftover serum will collected at the laboratory (CKCL LUMC) at each timepoint and each sample stored at -80°C in two aliquots. In addition urine samples will be collected at day C-1, day of treatment (T) and the day after treatment (T+1) for reasons of comparison with serum samples. All samples will be analyzed by NMR spectroscopy and mass spectrometry upon completion of the study. (Nevedomskaya 2011)
In collaboration with P. Crompton (NIAID, NIH, Rockville USA) and E Kirkness (J. Craig Venter Institute, Rockville, USA), RNA-seq technology will be used to determine RNA expression and processing differences between samples at day -1 (pre-challenge), day 5, day 6, day 9, day of treatment (T) and day 35 (follow-up).
.This will be performed on left-over blood collected for MLPA analysis, additional blood sampling is not required. This analysis will generate valuable knowledge on processes involved in protective immunity against malaria.

6.2 Randomisation, blinding and treatment allocation

There is no randomisation procedure in this study. A maximum of 25 volunteers (20 previously immunized and 5 new controls) will be exposed to 5 infected mosquitoes with NF135.C10 parasites. (See section 6.3.3. for additional information)
Blood samples are taken by medical staff not involved in the reading of blood smears. Thick smear readers and laboratory personal will be blinded. To prevent recognition by laboratory staff of previous ZonMw1 study volunteers all volunteers will receive a new study number. Samples taken from these volunteers are always blinded with respect of volunteer identity.

6.3 Study procedures

All volunteers will be closely followed as outpatients. For the duration of intensive monitoring (day 5 post-challenge until day 3 after treatment) they will be required to stay in the study hotel, close to the Leiden University Medical Centre. For the hotel period, volunteers are allowed to leave the hotel for daily activities, but will be required to stay at the hotel overnight for every night. They will receive breakfast at the hotel and one warm meal a day at the hospital canteen. Hotel and meal costs will be paid by the investigators. From day 5 till day 6 after challenge volunteers will visit the trial centre once a day. From day 7 till day 15 after challenge, volunteers will visit the trial centre two times a day. Since the likelihood of thick smear positivity drops significantly after the 15th day, the visit frequency is reduced to once daily from day 16 till day 21 after challenge. Day 22 till 24, during Malarone treatment, volunteers visit the trial centre twice a day. Volunteers are instructed to call the trial physicians at any time if they experience symptoms or complaints. The trial physician can decide to initiate additional diagnostics or treatment at all times. Table 6.3.6 shows the study procedures per visit.

Before the screening visit, volunteers will be asked to complete an application form that includes a questionnaire regarding their health (section F1b). All volunteers must consent with an HIV, hepatitis B, hepatitis C and toxicology test at screening. Female volunteers will be subjected to a urine pregnancy test at screening and the day before the challenge. All volunteers will repeat the urine toxicology test the day before challenge. At almost all visits, volunteers are subjected to blood withdrawal by venapuncture.
All volunteers are asked to complete a diary (section F2).

The challenge with mosquito bites will be performed at the Central Animal Laboratory Facility, of the RUNMC, Nijmegen, Netherlands. The biological safety parameters will be measured on serum samples at the central laboratory of the Leiden University Medical Centre. Assessment of blood slides will be performed by trained technicians of the department of Medical Microbiology of the Leiden University Medical Centre (LUMC). The immunological assessment will be performed at both sites.

6.3.1 Mosquito challenge

On the challenge day, all volunteers will be exposed to 5 infectious mosquitoes. The infection will be performed by placing a box containing mosquitoes on the forearm of the volunteer. (For more information concerning the production of mosquitoes, please refer to section 'Mosquito preparation' under 6.3.3). Mosquito feeding will be allowed for 10 minutes. Volunteers will receive a local treatment (tripelennamine crème) for mosquito bites and will be observed for 15 minutes after the feed. Directly after the feed, the mosquitoes will be dissected by a technician of the mosquito unit. This will be done to assure the presence of sporozoites in the salivary glands of the mosquitoes. Exposure will be repeated until the exact number of infected mosquito bites has been reached.
As long as there are volunteers present in the mosquito unit, there will be supervision of one of the clinical investigators. Another clinical investigator will then be on call, in case of emergency. Emergency aid kits will be present and readily available at any location, whenever there are volunteers present.

6.3.2 Treatment with Malarone®

All volunteers will be treated with Malarone® based on the following criteria:
1. Positive thick blood smear during regular visits
2. Complaints of malarial infection and positive thick blood smear
3. By decision of study doctor or the safety monitor
4. On request of the volunteer
5. On day 21 post challenge, if the volunteer has remained thick blood smear negative
6. When hs Troponine T (Roche) > 0.1 µg/ml or on recommendation of the cardiologist
7. When thrombocytes < 75 x 10^9/l
8. Dependent on values for LDH, D-dimer, ADAMTS13 and fragmentocytes according to the following scheme:

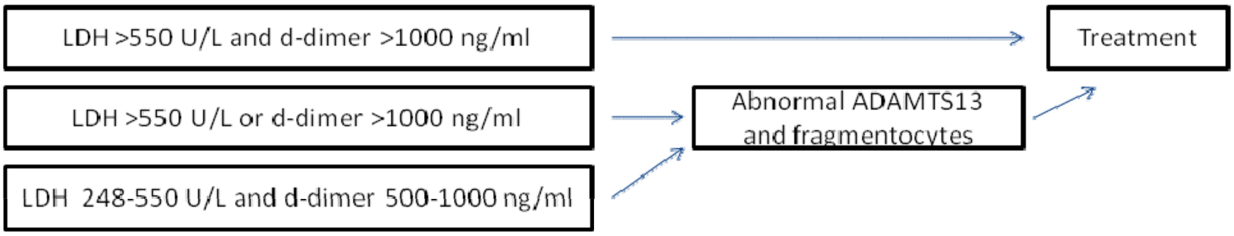

The treatment will consist of the drug Malarone® (atovaquon/proguanil). Dosing will be as follows: once daily 4 tablets of 250/100mg, during three days according to Dutch SWAB guidelines. This drug has been chosen because of its fast clinical response and the few side-effects. Furthermore, it has not been reported to have any cardiac related side-effect. During

treatment, complaints of malaria infection will be treated symptomatically. In addition to specific treatment with Malarone®, symptomatic treatment will be administered at the discretion of the attending physician.

Volunteers will not be admitted to the hospital during this study, unless the study doctors or the safety monitor deem it necessary, or on request of the volunteer.

D-dimer serum concentrations will be assessed by STA-R (Roche, Almere, The Netherlands). STA-R is a fully automated open random access coagulation analyser for clotting, chromogenic and immunological latex based coagulation tests. D-dimer concentrations are determined according to the manufacturer's instructions using an immunoturbidimetrical method, and are expressed in ng/mL (lower detection level 220 ng/mL upper detection level 5000 ng/mL).

6.3.3 Mosquito preparation

The culture of parasites and infection of mosquitoes has been a routine procedure for over 10 years now in the Malaria Unit of the Central Animal Facility of the RUNMC, Nijmegen.
The isolate used in this study will be the NF135.C10 isolate.
The field isolate NF135.C10 was obtained from a Dutch traveller to Cambodia diagnosed with Pf malaria in February 1993 and is isolated in the Radboud University Nijmegen. The isolate was adapted to continuous culture and selected for stable gametocyte production and infectiousness to mosquitoes. In August 2008, the strain was re-cultured from a cryopreserved batch and the isolate was cloned. NF135.C10 is sensitive for atovaquone, proguanil, arthemeter and lumefantrine.

RPMI-1640 medium with 10% serum and 5% haematocrit red blood cells. Both the serum and the red blood cells are obtained from the Nijmegen department of the Sanquin Bloedbank region Zuid-Oost, which is negative for malaria and seronegative for HIV, HBsAg, HCV, HTLV I+II and Lues. The cultures are checked for bacterial contamination, for mycoplasma and for blood transmitted diseases (HIV, HBV, HCV, HTLV 1+2).
To produce infectious gametocytes, the asexual parasites will be cultured in vitro. After 14 days of culture, the sexual stage parasites are obtained and prepared for feeding to 3-5 days old, and laboratory cultured *Anopheles stephensi* mosquitoes via a "membrane feeder". The percentage infected mosquitoes will be assessed after 7-10 days and after 14-17 days (see Product Information, D2a).

6.3.4 Blood sampling

During the study, blood samples will be drawn, for screening, safety and research purposes. The blood sampling schedule in the flow-chart section 6.3.6 shows the maximum amounts of blood that will be drawn. The following safety and screening tests will be performed:

- Haematology: Full Blood Count and differentiation of white cells, at V1 (screening), V2 (inclusion), daily during challenge period (day 5-21) until 3 days after treatment and at day 28 and 35.

- Biochemistry: Sodium, Potassium, Creatinin, ureum, ASAT, ALAT, AF, γGT, total bilirubin, at V1 (screening), V2 (inclusion), day T+2 and day 35. Glucose and total cholesterol are only for screening purposes.
- Highly sensitive Troponin T, d-dimer and LDH at V1 (screening), daily during challenge period (day 5-21) until 3 days after treatment.
- Fragmentocytes and ADAMTS13 if there are abnormal results for d-dimer and LDH according to the scheme in section 6.3.2.

Over a period of 35 days 500ml of blood will be withdrawn from the volunteers. This resembles the maximum amount of blood a volunteer could donate at a blood bank in the same period. Around 300ml, from the mentioned 500ml, is for safety purposes. The remaining 200ml is for secondary endpoints and immunological analysis. Logistical arrangements have been made to reduce the total amount of blood being withdrawn. For example out of a 3ml EDTA tube first a thick film is made and afterwards the remaining blood is used for PCR. Blood withdrawn for safety is taken in the smallest volume tubes available on the market. Maximum effort is made to minimize the amount of blood withdrawn from volunteers for safety reasons and at the same time optimizing the extraction of information and analysis from the withdrawn blood.

6.3.5 Case report forms and data collection

All data collected by the investigator is reported in case report forms. These forms, together with the investigator's notes are considered source data. Since all subjects will be healthy, there is no medical file for the study subjects, with exception of the medical file in case of adverse events/reactions resulting in a medical consultation or hospitalization. In this case the medical file will be considered as the source data. The diaries, produced by the study volunteers are also considered source data. They will be kept as source document in the investigator clinical file.

6.3.6 Flow chart trial procedures

	**Screening visit**	**Inclusion**	**Challenge**								
	Visits depending on day of thick smear positivity			Fixed visits							
**Visit Number**	V1	V2	V3	V4-5	V6-23	V24-28	V29	V30-35	V36	V37	V38
**Trial timeline (D=day)**	± D-14	D-1	D0	D5-6	D7-15	D16-20	TD[8] or D21	TD+1-3	D28	D35	D140
**Frequency of visits**	1x	1x	1x	1x	2x	1x	1x	2x	1x	1x	1x
**Eligibility criteria + informed consent**	X										
**Demographic data, Medical history**	X										
**Physical examination**	X	X		X[1]	X[1]	X[1]	X	X[1]	X[1]	X[1]	X[1]
**ECG**	X										
**Vital signs**	X	X		X	X	X	X	X	X	X	X
**Challenge with 5 infected mosquitoes**			X								
**Collecting (serious) adverse events**		X	X	X	X	X	X	X	X	X	X
**Malarone® treatment**							X	X			
**Haematology tests[2,11] (3 ml)**	X	X		X*	X**	X*	X*	X*	X*	X*	X*
**Biochemistry tests[3] (3 ml)**	X	X						X (TD+2)		X	
**Glucose (2 ml)**	X										
**Serology[4] (6 ml)**	X										
**Parasitology[5] (3 ml)**		X		X*	X**	X*	X*	X (DT+3)[9]	X*	X	
**Cellular/humoral immune response[6]**		X								X	X
**Metabolomics and dc-RT-MLPA[7,11]**		X[7,12]		X[7]	X[7]		X[7,12]	X[12]		X[7]	
**Pregnancy and toxicology urine test**	X	X									
**hs Trop T, LDH, d-dimer**	X			X	X	X	X	X			
**Reviewing diaries**		X		X	X	X	X	X	X	X	

**Safety report**	X[10]	X

D = day, V = visit, TD = treatment day
*: once daily; **: twice daily

1: On indication
2: Hb, haematocrit, MCV, MCH, MCHC, platelets, WBC + differentiation
3: Creatinin, urea, sodium, potassium, bilirubin, AF, γGT, ASAT, ALAT, additional at screening: total cholesterol + glucose
4: HIV, HBV, HCV, Pf
5: Thick smear, PCR
6: Plasma collected in CPT tubes will be stored for cellular and humoral assays
7: Immunological (dcRT-MLPA) en Metabolomic (serum) evaluation on day before challenge, day 5, 6, 9, day of treatment and day 35
8: TD: day of thick smear positivity and/or start treatment Malarone®
9: Thick smear at TD+3, if not negative treatment until twice negative
10: Safety evaluation on day 8, 13, 35 and 140.
11: Subset analysis: On day 5, 6, 9, day of treatment and day 35 1 ml of blood will be taken out of the EDTA tube for subset analysis. Results are to be related to dcRT-MLPA analysis.
12. Metabolomic (urine) evaluation on day before challenge (C-1), day of treatment (T) and day after treatment (T+1).

6.4 Withdrawal of individual subjects

Subjects can leave the study at any time for any reason if they wish to do so without any consequences. The investigator can decide to withdraw a subject from the study for urgent medical reasons.
Volunteers can be withdrawn from the study procedures for the following reasons:

- Any serious adverse event
- Any adverse event that, according to clinical judgment of the investigator, is considered as a definite contra-indication to proceeding with the study procedures.
- The use of concomitant, chronic medication active on the immune system (steroids, immunosuppressive agents)
- Pregnancy
- Withdrawal of informed consent by volunteer
- Completely lost to follow-up

6.5 Follow-up of subjects withdrawn from treatment

If a subject fails to appear for a follow-up examination, extensive effort (i.e. documented phone calls and certified mail) will be undertaken to locate or recall the volunteer and at least to determine health status. These efforts will be documented in the subject's CRF and source documents. In the event that a volunteer discontinues the study for any reason, the volunteer will be required to complete all safety follow-up as appropriate, as determined by the principle investigator and the safety monitor. All volunteers will be included in the safety analysis.

6.6 Premature termination of the study

The study may be discontinued for the following reasons:

- On advice of the safety monitor
- On advice of the DSMB
- On advice of the investigators
- On advice of the IRB
- Withdrawal of Informed consent by the volunteer

The safety monitor, DSMB, IRB or investigators may decide to put the study on hold based on adverse events, pending discussion with the IRB/DSMB/safety monitor/investigators. Following discussion, it may be decided to terminate the study. Safety reporting procedures are described in section 7.

7. SAFETY REPORTING

7.1 Section 10 WMO event

In accordance to section 10, subsection 1, of the WMO, the investigator will inform the subjects and the reviewing accredited METC if anything occurs, on the basis of which it appears that the disadvantages of participation may be significantly greater than was foreseen in the research proposal. The study will be suspended pending further review by the accredited METC, except insofar as suspension would jeopardise the subjects' health. The investigator will take care that all subjects are kept informed.

7.2 Adverse and serious adverse events

7.2.1 Adverse events

Adverse events are defined as any undesirable experience occurring to a subject during the study, whether or not considered related to trial. An AE can therefore be any unfavourable and unintended sign (including an abnormal laboratory finding), symptom, or disease (new or exacerbated) temporally associated with the use of a medicinal product or study intervention. AEs may include events that occur as a result of protocol-mandated procedures (i.e. invasive procedures, modification of subject's previous therapeutic regimen). All adverse events reported spontaneously by the subject or observed by the investigator or his staff will be recorded. Abnormal laboratory findings (e.g., clinical chemistry, haematology, urinalysis) or other abnormal assessments that are judged by the investigator to be clinically significant will be recorded as AEs or SAEs if they meet the definition. The investigator will exercise his or her medical and scientific judgment in deciding whether an abnormal laboratory finding or other abnormal assessment is clinically significant.

If there are any complaints not typical for malaria infection, such as for example chest pain or dyspnoea, the volunteer will be evaluated immediately by a qualified clinician using the appropriate clinical tools (eg. ECG or measurement of cardiac enzymes) according to hospital care in LUMC.

7.2.2 Serious adverse events

A serious adverse event is any untoward medical occurrence or effect that at any dose:

- results in death;
- is life threatening (at the time of the event);
- requires hospitalisation or prolongation of existing inpatients' hospitalisation;
- results in persistent or significant disability or incapacity;
- is a congenital anomaly or birth defect;
- is a new event of the trial likely to affect the safety of the subjects, such as an unexpected outcome of an adverse reaction, lack of efficacy of an IMP used for the treatment of a life threatening disease, major safety finding from a newly completed animal study, etc.

All SAEs will be reported through the web portal ToetsingOnline to the IRB that approved the protocol, within 15 days after the sponsor has first knowledge of the serious adverse reactions. SAEs that result in death or are life threatening will be reported expedited. The expedited reporting will occur not later than 7 days after the responsible investigator has first knowledge of the adverse reaction. This is for a preliminary report with another 8 days for completion of the report.

7.2.3 Adverse Event Data Collection

Safety assessments will be performed, and recorded by the investigators. All adverse events/reactions (solicited and unsolicited), observed by the investigators or by the subject, will be accurately documented in the case report form by the investigators. For each event/reaction the following details will be recorded:

1. description of the event(s)/reaction(s)
2. date and time of occurrence
3. duration
4. intensity
5. relationship with the intervention
6. action taken, including treatment
7. outcome

In addition, symptoms will be ranked as (1) mild, (2) moderate, or (3) severe, depending on their intensity. All adverse events except fever will be judged for their intensity according to the following scale:

- Mild (grade 1): awareness of symptoms that are easily tolerated and do not interfere with usual daily activity
- Moderate (grade 2): discomfort that interferes with or limits usual daily activity
- Severe (grade 3): disabling, with subsequent inability to perform usual daily activity, resulting in absence or required bed rest

For fever, the following scale will be used:

- Mild (grade 1): 37.5 - 38.0°C
- Moderate (grade 2): > 38.0 to 39.0°C
- Severe (grade 3): > 39.0°C

If an AE changes in frequency or intensity during the specified reporting period, the previous description of the AE will be corrected.

When an AE/SAE occurs, it is the responsibility of the investigators to review all documentation (e.g., hospital progress notes, laboratory, and diagnostics reports) related to the event. The investigators will then record all relevant information regarding an AE/SAE on the CRF or SAE Report Form, respectively.

The investigator will attempt to establish a diagnosis of the event based on signs, symptoms, and/or other clinical information. In such cases, the diagnosis should be documented as the AE/SAE and not the individual signs/symptoms.

7.2.4 Assessment of causality

The investigators are obligated to assess the relationship between study procedures and the occurrence of each AE/SAE. The investigators will use clinical judgment to determine the relationship. Alternative causes, such as natural history of the underlying diseases, concomitant therapy, other risk factors and the temporal relationship of the event to the challenge will be considered and investigated. The relationship of the adverse event with the study procedures will be categorized as:

Probable	An adverse event that follows a reasonable temporal sequence from the challenge procedure and cannot be reasonably explained by the known characteristics of the subject's clinical state.
Possible	An adverse event for which insufficient information exists to indicate a high improbability that the event is related to the study procedure.
Not related	An event for which sufficient information exists to indicate that the etiology is unrelated either because of the temporal sequence of events or because of the subject's clinical state or other therapies

7.2.5 Follow-up of adverse events

All adverse events will be followed until they have abated, or until a stable situation has been reached. Depending on the event, follow up may require additional tests or medical procedures as indicated, and/or referral to the general physician or a medical specialist.

Investigators will follow-up subjects:
-With SAEs or those withdrawn from the study as a result of an AE: until the event has resolved, subsided, stabilized, disappeared, the event is otherwise explained, or the subject is lost to follow-up.
-With other non-serious AEs: until the subject has completed the study or is lost to follow-up.

Clinically significant laboratory abnormalities will be followed up until they have normalized, or until an alternative explanation, that is not related to the study has been provided. Additional information (including but not limited to laboratory results) relative to the subsequent course of such an abnormality will be made available to the safety monitor.

7.3 Local Safety Monitor and Data Safety Monitoring Board (DSMB)

For this study, a local safety monitor is appointed who is based in the Leiden University Medical Centre and will be involved in the review of severe and serious adverse events and volunteer safety. He is an experienced clinician qualified to evaluate safety data from clinical studies with malaria infections. He is independent of the investigator team. His main responsibility will be the assessment of the events and recommendation regarding halting further immunizations or challenge. Furthermore, an independent Data Safety Monitoring Board has been appointed.

7.3.1 Review of Safety Data by the Safety Monitor and DSMB

Before challenge, the safety data will be discussed in a meeting between the study investigator and the Safety Monitor. A safety report including a list of all reported adverse events and any safety laboratory values outside the normal ranges will be prepared on day 8, 13, 35 and 140 after challenge. These reports will be prepared by the Principal Investigator and sent to the Safety Monitor and all clinical investigators involved. The Safety Monitor will review the safety data within 2 working days and if warranted instruct the site to withdraw or treat individual subjects and/or suspend further study procedures. In addition, safety data on day 35 will be assessed by the Data Safety Monitoring Board. Responsibilities of the Data Safety Monitoring

Board are described in the DSMB Charter (section K5).
All serious adverse events will be reported by the Principal Investigator to the Safety Monitor, the Sponsor and the CCMO within 24 hours. Any highly sensitive troponin T value greater than 0.03 μg/L will be reported to the Safety Monitor within 24 hours. Any laboratory values leading to immediate malaria treatment will be reported to the Safety Monitor within 24 hours.
The advice(s) of the DSMB will be notified upon receipt by the sponsor to the CCMO that approved the protocol. With this notification a statement will be included indicating whether the advice will be followed.

8. STATISTICAL ANALYSIS

All challenged volunteers will be included in the intention-to-treat analysis. Complete protection is defined as negative thick smears till day 21 after challenge infection.
As the primary analysis of the trial, we will compare previously immunized individuals with controls, taking presence or absence of complete protection as the endpoint. Difference in proportions between groups will be assessed using Fisher's exact test.
In the statistical analysis group 1 (previously immunized) volunteers previously protected and unprotected individuals will be analyzed on an individual basis.

Development of parasitaemia will be compared between groups using the statistical model, which will provide the estimated number of infected hepatocytes and the parasite multiplication rate per volunteer (Hermsen 2001). Differences between mean number of infected hepatocytes and parasite multiplication rate will be assessed by two-tailed student's t-test (if comparing two groups) or a one-way ANOVA (if comparing more than two groups) or non-parametric equivalents.
The secondary endpoints will be assessed by comparing mean values between the groups using either a two-tailed student's t-test (if comparing two groups) or a one-way ANOVA (if comparing more than two groups) or non-parametric equivalents, paired if pre-challenge values are compared with post challenge values, unpaired if comparisons are made between groups.
For discrete variables (e.g. the number of positive tick smears in a group, the number of positive assays), the chi-squared test or Fisher's exact test will be used (two-tailed).
All adverse events for each volunteer will be tabulated. Adverse events will be analyzed by calculating the proportion of volunteers in each group who report mild, moderate or severe adverse events. The frequency of signs and symptoms will be compared between groups with the chi-square test.

9. ETHICAL CONSIDERATIONS

9.1 Regulation statement

This study will be conducted in accordance with the latest Seoul revision of the Declaration of Helsinki (section K6b), the Medical Research Involving Human Subjects Act (WMO), the ICH Good Clinical Practice, and local regulatory requirements.
The investigators shall be responsible for obtaining Ethics Committee(s) approval (IRB) of the protocol and any subsequent amendments in compliance with local law before the start of the study.

9.2 Recruitment and consent

As soon as the study is approved by the CCMO, advertisements will be placed on different University campuses in the West of the Netherlands and other public places as well as on the intranet of the institutions. The investigators will be responsible for providing adequate verbal and written information regarding the objectives and procedures of the study, the potential risks involved and the obligations of the volunteers. Volunteers will be informed that they will not gain health benefits from this study. Trainees or other students who might be dependent on the investigators or the study group will not be included in the study. After free discussion with the investigator, the volunteer will be given sufficient time to consider participation. Obtaining informed consent and screening of volunteers will not start until full approval is obtained from the CCMO. On the screening visit written informed consent is obtained from the volunteer. A sample volunteer information letter containing this information and informed consent form can be found in sections E1 and E2.
Volunteers will be informed that they can withdraw their informed consent at any time during the study.

9.3 Benefits and risks assessment, group relatedness

Two major areas of ethical concerns are contained within this proposal, namely the use of blood from humans and the use of human volunteers for controlled human malaria infections. All partners in this proposal are aware of and follow the relevant national and international rules and regulations as they pertain to access to material of human origin and clinical research. International agreements such as the Helsinki declaration and the Convention of the Council of Europe on Human Rights and Biomedicine will be observed and respected.

9.3.1 Ethical aspects concerning the production of *P. falciparum* infected mosquitoes

The human blood used in this study is declassified from screened healthy blood donors from the hospital blood bank and parasites are cultured with serum. Continuous culture of drug sensitive *Plasmodium falciparum* has been routine over the past 2 decades of the Central Animal Facility of the RUNMC. All strains originate from patient material. All culture material is checked for bacterial contamination, mycoplasma and for blood transmitted diseases (HIV, HBV, HCV, HTLV 1+2).

9.3.2 Ethical aspects concerning the use of human volunteers

Infection of human volunteers with malaria has been carried out over several decades including therapeutic use as treatment for neurosyphilis and later for vaccine evaluation. The ability to carry out this type of work is largely based on the relatively low morbidity and (in more recent times) the lack of mortality seen in these studies. The occurrence of a cardiac event in a volunteer participating in a combined phaseI/IIa malaria vaccine trial in Nijmegen has raised intense discussion on the safety of malaria challenge trials with respect to cardiac events. Based on recommendations of the CCMO and an External Scientific Advisory Committee to the European Malaria Vaccine Development Association, this malaria challenge trial protocol has been adjusted (section 1.5).

The only reliable and convincing way to obtain information on the capacity of potential vaccine strategies to induce protection will be testing in human subjects. The compelling need for a malaria vaccine should be balanced to the potential risks and discomforts of the volunteers. Explorative studies of a human immunization model for pre-erythrocytic malaria vaccines is of paramount importance and a potentially powerful tool in the difficult decision making process of bringing vaccine candidates into endemic countries

The study will be undertaken in accordance with good clinical practice, according to the standards defined in the EEC directive 91/507/EEC, and in the Directive on Good Clinical Practice in Clinical Trials (ICH GCP, 75/318/EEC, January 1997) and under the principles of the Declaration of Helsinki (section K6b); ethical permission will be sought from the CCMO the Netherlands.

9.4 Compensation for injury

The sponsor has a liability insurance which is in accordance with article 7, subsection 6 of the WMO, covered by the RUNMC insurance for human research. This is provided by Akkermans van Elten Assurantiën BV, Postbus 181, 6660 AD Elst. Volunteer insurance text is provided in section E4.

9.5 Incentives

Volunteers will receive a maximum of 1000 Euros in compensation fee; travel expenses will not be additionally reimbursed. This amount of money is reasonable and in line with Dutch common practice. In case of unexpected medical complications, there will be state-of-the-art medical treatment at full costs covered by the insurance of Radboud University Nijmegen Medical Centre.

10. ADMINISTRATIVE ASPECTS AND PUBLICATION

10.1 Handling and storage of data and documents

All parties agree to adhere to the principles of medical confidentiality in relation to Clinical Study Subjects involved in the Clinical Study. Neither party shall disclose the identity of Clinical Study Subjects to third parties without prior written consent of the Clinical Study Subject.

All serum samples, or other volunteer material will be labelled with the volunteer study identification number. The samples will not be labelled with volunteer names or birth dates.

Samples will be stored for 15 years.

10.2 Amendments
Amendments are changes made to the research after a favourable opinion by the accredited METC has been given. All amendments will be notified to the CCMO.

10.3 Annual progress report
The sponsor will submit a summary of the progress of the trial to the CCMO once a year. Information will be provided on the date of inclusion of the first subject, numbers of subjects included and numbers of subjects that have completed the trial, serious adverse events/ serious adverse reactions, other problems, and amendments.

10.4 End of study report
The investigator will notify the CCMO of the end of the study within a period of 90 days. The end of the study is defined as the last volunteer's last visit. In case the study is ended prematurely, the investigator will notify the CCMO, including the reasons for the premature termination. Within one year after the end of the study, the investigator/sponsor will submit a final study report with the results of the study, including any publications/abstracts of the study, to the CCMO.

10.5 Public disclosure and publication policy
The final report will be prepared by the investigators in collaboration with the Radboud University Nijmegen Medical Centre (RUNMC), department of Medical Microbiology (MMB) representative. It will be signed by the coordinating and the principal investigator. The protocol and data derived from the trial are the exclusive property of the RUNMC-MMB.

All rights, title, and interests in any inventions, know-how or other intellectual or industrial property rights which are conceived or reduced to practice by site staff during the course of or as a result of the study are hereby assigned to the RUNMC-MMB.

Any publication or presentation related to the trial must be approved by the RUNMC-MMB representative and principle investigator before submission of the manuscript. After publication of the results of the trial, any participating centre may publish or otherwise use its own data provided that any publication of data from the trial gives recognition to the trial group. Either centre must have the opportunity to review the proposed abstract, manuscript or presentation at least 14 days before submission for publication/presentation.

Any information identified as confidential must be deleted prior to submission, it being understood that the results of this trial are not to be considered confidential.

10.6 List of Publication and Authorship
The authorized persons as an author of the publication(s) are those who have contributed to the protocol and/or to the analysis of the data, and whose names are listed on the flyleaf. According to the main topic of the publication, the first author will be the greatest contributing investigator (or biological evaluator).

11. REFERENCES

Abdulla S, Oberholzer R, Juma O, Kubhoja S, Machera F, Membi C, et al. *Safety and immunogenicity of RTS,S/AS02D malaria vaccine in infants*. The New England journal of medicine. 2008 Dec 11;359(24):2533-44.

Agnandji ST, Lell B, Soulanoudjingar SS et al. *First results of phase 3 trial of RTS,S/AS01 malaria vaccine in African children*. N Engl J Med. 2011 Nov 17;365(20):1863-75. Epub 2011 Oct 18.

Beeson JG, Osier FH, Engwerda CR. *Recent insights into humoral and cellular immune responses against malaria.* Trends in parasitology. 2008 Dec;24(12):578-84.

Belnoue E, Costa FT, Frankenberg T, Vigario AM, Voza T, Leroy N, et al. *Protective T cell immunityagainst malaria liver stage after vaccination with live sporozoites under chloroquine treatment.* J Immunol. 2004 Feb 15;172(4):2487-95.

Breman JG, Plowe CV. *A malaria vaccine for control: more progress*. The Journal of infectious diseases. 2009 Aug 1;200(3):317-20.

Chulay JD, Schneider I, Cosgriff TM, Hoffman SL, Ballou WR, Quakyi IA, et al*. Malaria transmitted to humans by mosquitoes infected from cultured Plasmodium falciparum.* The American journal of tropical medicine and hygiene. 1986 Jan;35(1):66-8.

Church LW, Le TP, Bryan JP, Gordon DM, Edelman R, Fries L, et al. *Clinical manifestations of Plasmodium falciparum malaria experimentally induced by mosquito challenge.* The Journal of infectious diseases. 1997 Apr;175(4):915-20.

Collins WE, Contacos PG. *Immunization of monkeys against Plasmodium cynomolgi by X-irradiated sporozoites.* Nature: New biology. 1972 Apr 12;236(67):176-7.

Doolan DL, Dobano C, Baird JK. *Acquired immunity to malaria*. Clinical microbiology reviews. 2009 Jan;22(1):13-36.

Epstein JE, Giersing B, Mullen G, Moorthy V, Richie TL. *Malaria vaccines: are we getting closer?* Current opinion in molecular therapeutics. 2007 Feb;9(1):12-24.

Epstein JE, Rao S, Williams F, Freilich D, Luke T, Sedegah M, et al. *Safety and clinical outcome of experimental challenge of human volunteers with Plasmodium falciparum-infected mosquitoes: an update. The Journal of infectious diseases.* 2007 Jul 1;196(1):145-54.

Hermsen CC, Telgt DS, Linders EH, van de Locht LA, Eling WM, Mensink EJ, et al. *Detection of Plasmodium falciparum malaria parasites in vivo by real-time quantitative PCR.* Molecular and biochemical parasitology. 2001 Dec;118(2):247-51.

Hermsen CC, de Vlas SJ, van Gemert GJ, Telgt DS, Verhage DF, Sauerwein RW. *Testing vaccines in human experimental malaria: statistical analysis of parasitemia measured by a quantitative real-time polymerase chain reaction.* The American journal of tropical medicine and hygiene. 2004 Aug;71(2):196-201.

Hoffman SL, Goh LM, Luke TC, Schneider I, Le TP, Doolan DL, et al. *Protection of humans against malaria by immunization with radiation-attenuated Plasmodium falciparum sporozoites.* The Journal of infectious diseases. 2002 Apr 15;185(8):1155-64.

Hoffman SL, Billingsley PF, James E, Richman A, Loyevsky M, Li T, et al. *Development of a metabolically active, non-replicating sporozoite vaccine to prevent Plasmodium falciparum malaria*. Human vaccines. 2010 Jan 21;6(1).

Nevedomskaya E, Mayboroda OA, Deelder AM. *Cross-platform analysis of longitudinal data in metabolomics*. Molecular Biosystems 2011, DOI: 10.1039/c1mb05280b

Nicholson, Jeremy K, Lindon John C. *Spectoscopic and statistical techniques for information recovery in metabolomics.* Annu. Rev. Anal. Chem. 2008: 1:45-69

Nieman AE, de Mast Q, Roestenberg M, Wiersma J, Pop G, Stalenhoef A, et al. *Cardiac complication after experimental human malaria infection: a case report.* Malaria journal. 2009;8:277.

Pombo DJ, Lawrence G, Hirunpetcharat C, Rzepczyk C, Bryden M, Cloonan N, et al. *Immunity to malaria after administration of ultra-low doses of red cells infected with Plasmodium falciparum.* Lancet. 2002 Aug 24;360(9333):610-7.

Roll back malaria progress and impact series September 2011

Roestenberg M, McCall M, Hopman J, Wiersma J, Luty AJ, van Gemert GJ, et al*. Protection against a malaria challenge by sporozoite inoculation.* The New England journal of medicine. 2009 Jul 30;361(5):468-77.

Roestenberg M, Teirlinck AC, McCall MB, Teelen K, Makamdop KN, Wiersma J, Arens T, Beckers P, van Gemert G, van de Vegte-Bolmer M, van der Ven AJ, Luty AJ, Hermsen CC, Sauerwein RW. *Long-term protection against malaria after experimental sporozoite inoculation: an open-label follow-up study.* Lancet. 2011 May 21;377(9779):1770-6. Epub 2011 Apr 22.

Sauerwein RW, Bijker EM, Richie TL. *Empowering malaria vaccination by drug administration*. Curr Opin Immunol. 2010 Jun;22(3):367-73.

Targett GA, Greenwood BM. *Malaria vaccines and their potential role in the elimination of malaria*. Malaria journal. 2008;7 Suppl 1:S10.

Verhage DF, Telgt DS, Bousema JT, Hermsen CC, van Gemert GJ, van der Meer JW, et al. *Clinical outcome of experimental human malaria induced by Plasmodium falciparum-infected mosquitoes.* The Netherlands journal of medicine. 2005 Feb;63(2):52-8.

Walther M, Jeffries D, Finney OC, Njie M, Ebonyi A, Deininger S, Lawrence E, Ngwa-Amambua A, Jayasooriya S, Cheeseman IH, Gomez-Escobar N, Okebe J, Conway DJ, Riley EM. *Distinct roles for FOXP3 and FOXP3 CD4 T cells in regulating cellular immunity to uncomplicated and severe Plasmodium falciparum malaria*. PLoS Pathog. 2009 Apr;5(4):e1000364. Epub 2009 Apr 3.

WHO Malaria report 2011
