# Supplementary material for: Heterologous Protection against Malaria after Immunization with Plasmodium falciparum Sporozoites
Source: PLoS One. 2015 May 1;10(5):e0124243. doi: 10.1371/journal.pone.0124243 (PMC4416703; doi:10.1371/journal.pone.0124243)
Supplement: S1 Flow Diagram — (DOC) [file pone.0124243.s003.doc]

**
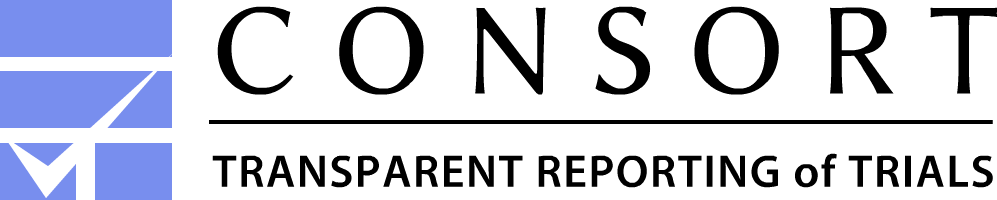
**

**CONSORT 2010 Flow Diagram**

**Allocation**

**Analysis**

**Follow-Up**

**Enrollment**

Assessed for eligibility (n=26)

Excluded (n=4)

♦  Not meeting inclusion criteria (n=0)

♦  Declined to participate (n=1)

♦  Other reasons (n=3)

Analysed (n=16)
♦ Excluded from analysis (n=0)

Lost to follow-up (give reasons) (n=0)

Discontinued intervention (n=1) (reason: positive urine toxicology)

Allocated to intervention (n=17)

♦ Received allocated intervention (n=17)

♦ Did not receive allocated intervention (n=0)

Lost to follow-up (give reasons) (n=0)

Discontinued intervention (n=0)

Allocated to control (n=5)

♦ Received allocated intervention (n=5)

♦ Did not receive allocated intervention (n=0)

Analysed (n=5)
♦ Excluded from analysis (n=0)

**Non-Randomized** (n=22)
