# Supplementary material for: Heterologous Protection against Malaria after Immunization with Plasmodium falciparum Sporozoites
Source: PLoS One. 2015 May 1;10(5):e0124243. doi: 10.1371/journal.pone.0124243 (PMC4416703; doi:10.1371/journal.pone.0124243)
Supplement: S1 Methods — (DOCX) [file pone.0124243.s004.docx]

**Supplemental Methods**

## Sample size calculation

Our primary objective was to determine protection against a heterologous *P. falciparum* re-challenge after previous CPS immunized and challenged subjects on an individual basis.

A statistical model of parasitemia after mosquito infection has been developed, which is based on quantitative real-time PCR measurements of parasitaemia (Figure A) [[1](#_ENREF_1)].

**Number of parasites/ml determined**

**by QRT-PCR**

Figure A: Observed and predicted parasite densities of 5 representatives out of 15 individuals experimentally infected with *Plasmodium falciparum*. Markers represent observed number of parasites per ml blood based on the quantitative real-time PCR results. Observations of one individual have the same colour. Individual observations were continued until a standard blood smear was found positive, after which immediate treatment was provided. The best fitting curve for each individual is represented in the same colour as the corresponding markers.

We used our model to simulate observations for non-protected and protected experimentally infected human volunteers over time [[1](#_ENREF_1)]. Three sources of variation, as determined from previous testing of the model against data from 15 volunteers, were included in the simulations: (1) individual variation in level of infection (number of infected hepatocytes); (2) individual variation in timing of the first generation of ring forms after the hepatocyte stage; and (3) variation in measurement error. Previously estimated parameters for mean/variance of duration of presence/absence of ring forms and the multiplication factor between successive parasite cycles were pre-fixed. This quantitative analysis permits a detailed estimation of critical parameters in the parasite life cycle including duration of trophozoite maturation and multiplication of blood stages due to a remarkable consistent pattern of PCR positive parasitaemia before the blood slide becomes positive and treatment is required.

In this project, the main protective effects were expected at the level of the liver stage, but some protective mechanisms could operate on asexual blood forms. We made a power calculation for the minimal degree of asexual stage protection that could be shown with a group size of 5 volunteers. The effect of a 60% effective asexual stage protection was mimicked by a 60% reduction of the number of ring forms per hepatocyte and the multiplication factor. To perform a power analysis, such simulations were repeated 100 times. From this we concluded that testing 60% effective asexual stage protection using 5 individuals per group and α = 5% will give significant results in more than 90% of the experiments). When using 10 individuals per group, this will be >95%.

**References**

1. Hermsen CC, de Vlas SJ, van Gemert GJ, Telgt DS, Verhage DF, et al. (2004) Testing vaccines in human experimental malaria: statistical analysis of parasitemia measured by a quantitative real-time polymerase chain reaction. Am J Trop Med Hyg 71: 196-201.
